# A Method to Enrich Functional Human Paneth Cells in Induced Pluripotent Stem Cell-Derived Intestinal Organoids

**DOI:** 10.1016/j.jcmgh.2026.101769

**Published:** 2026-03-18

**Authors:** Shachi Patel, Monica S. Wagner, Olivia Bay, Christian E. Wong Valencia, Eliska Zgarbova, Cynthia I. Rodriguez, Daniel N. Leal, Michifumi Yamashita, Suzanne Devkota, Kathrin S. Michelsen, Stephan R. Targan, Robert J. Barrett

**Affiliations:** Cedars-Sinai Medical Center, Board of Governors Regenerative Medicine Institute, Los Angeles, California; Cedars-Sinai Medical Center, F. Widjaja Inflammatory Bowel Disease Institute, Los Angeles, California; Department of Pathology and Laboratory Medicine, Cedars-Sinai Medical Center, Los Angeles, California; Cedars-Sinai Medical Center, F. Widjaja Inflammatory Bowel Disease Institute, Los Angeles, California; Department of Medicine, Cedars-Sinai Medical Center, Human Microbiome Research Institute, Los Angeles, California; Department of Medicine and Biomedical Sciences, Cedars-Sinai Medical Center, Los Angeles, California; Cedars-Sinai Medical Center, F. Widjaja Inflammatory Bowel Disease Institute, Los Angeles, California; Department of Medicine and Biomedical Sciences, Cedars-Sinai Medical Center, Los Angeles, California; Cedars-Sinai Medical Center, Board of Governors Regenerative Medicine Institute, Los Angeles, California; Cedars-Sinai Medical Center, F. Widjaja Inflammatory Bowel Disease Institute, Los Angeles, California; Department of Medicine and Biomedical Sciences, Cedars-Sinai Medical Center, Los Angeles, California

**Figure:**
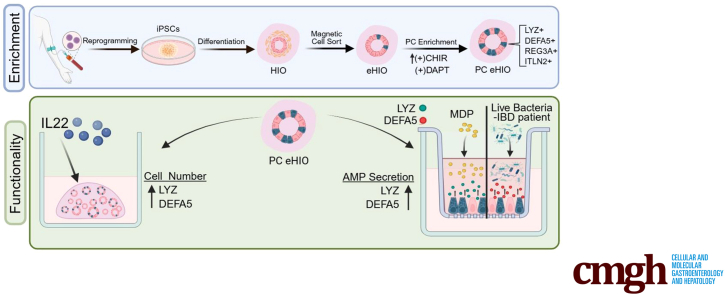

Paneth cells are a specialized intestinal epithelial subtype that reside in the crypt of the small intestine, and whose major function is to produce antimicrobial peptides (AMPs). Genetic variants in the *NOD2* and *ATG16L1* genes^1^^,^^2^ are associated with an altered Paneth cell phenotype but the confounding influences of the microbiome and mucosal immune system make it challenging to determine whether genetic variations or environmental influences are the cause of these altered phenotypes. Therefore, our goal was to develop a biologically responsive in vitro Paneth cell model that would permit an examination of both intrinsic and extrinsic influences in a controlled reductionist system.

We chose to utilize induced pluripotent stem cells (iPSCs) for the source of our human intestinal organoids (HIOs), as this is a donor cell type that can be obtained either from a simple blood draw^3^ or lymphoblastoid cell lines from numerous-well characterized biorepositories,^4^ and iPSC-derived HIOs have previously been shown to be representative of the small intestine.^5^ We directed a control iPSC line (CS03iCTR-n1) to HIOs as previously described,^6^ and the initial characterization of our 10d iPSC-derived HIOs revealed a complete absence of lysozyme+ cells. After 30 days, only 2 organoids in one series of experiments were found to be lysozyme+ but were negative for additional AMPs (Supplementary Figure 1*A*). Given the near absence of Paneth cells, we thus aimed to enrich our organoids for this cell type. Numerous attempts have been made to enrich for Paneth cells in various modalities such as murine organoids and human biopsy-derived intestinal organoids (Supplementary Table 1), and so to test a subset of these approaches, we first purified the epithelial cellular component of 10-day and 30-day HIOs, cultured them as epithelial-only HIOs (eHIOs), which could be serially passaged every 7 days, with the goal of examining such approaches (Supplementary Figure 1*B*). Given that we found that eHIOs from 10-day iPSC-derived HIOs again had no lysozyme+ cells, whereas those from 30-day iPSC-derived HIOs contained a small presence (Supplementary Figure 1*C*), we thus utilized eHIOs from the 30-day timepoint in all studies listed below.

Previous attempts to enrich for Paneth cells in the organoid modeling system generally utilized the Notch inhibitor, DAPT, and the GSK-inhibitor CHIR99021, and although this resulted in increased numbers of lysozyme+ cells (∼ 20-85%) in murine intestinal organoids, human biopsy-derived organoids were generally limited to increases in the expression of Paneth cell-related genes (Supplementary Table 1).Two studies attempted to enrich via interleukin (IL)-22 addition in biopsy-derived human intestinal organoids and this resulted in the presence of ∼1% to 15% Paneth cells (Supplementary Table 1). To assess both of these approaches in our iPSC-derived cells, we first modified our eHIO maintenance media and found the removal of the small molecules SB202190/A83-01 and increased concentration of CHIR99021 (2μM→3μM) (Methods and Supplementary Table 2) led to a significant increase in lysozyme+ cells from ∼2.5% to ∼10%, and although there was no significant increase with the addition of IL-22, there was a significant increase to ∼30% lysozyme+ cells with the addition of DAPT (Figure 1*A*). We then assessed for previously identified human Paneth cell-related genes^7^ and found that increased CHIR99021 and DAPT led to a significant increase in the expression of *DEFA5, DEFA6, PLA2G2A, REG3A*, and *ITLN2* mRNA (Figure 1*B*) as compared with those in maintenance media. We then confirmed via immunofluorescent stainings that these factors led to numerous cells within eHIOs to possess not only granulated lysozyme, but also granulated DEFA5, REG3A, and ITLN2 (Figure 1*C*), and transmission electron microscopy revealed the presence of electron dense granules (Figure 1*D*), all of which suggests the presence of an enriched Paneth cell population. Given that Notch inhibition can globally influence secretory differentiation, we also observed increased expression of both *CHGA* and *MUC2*, which suggests that both enteroendocrine and goblet cells are obtained also (Supplementary Figure 2*C*). Finally, to confirm that this protocol is not restricted to the aforementioned control iPSC line, we demonstrated that the addition of increased CHIR99021 and DAPT to eHIOs, generated from 2 other control iPSC lines, also gave enriched Paneth cell populations. Under ENC(3)+DAPT conditions, CS2GW3i eHIOs contained 45.8% LYZ^+^ and 17.5% DEFA5^+^ cells, whereas CS9EWPi eHIOs showed 33.6% LYZ^+^ and 11.5% DEFA5^+^ cells (Supplementary Figure 2*A*), and there was significantly upregulated *DEFA5*, *DEFA6*, *PLA2G2A*, and *REG3A* in both lines (Supplementary Figure 2*B*).

**Figure 1:**
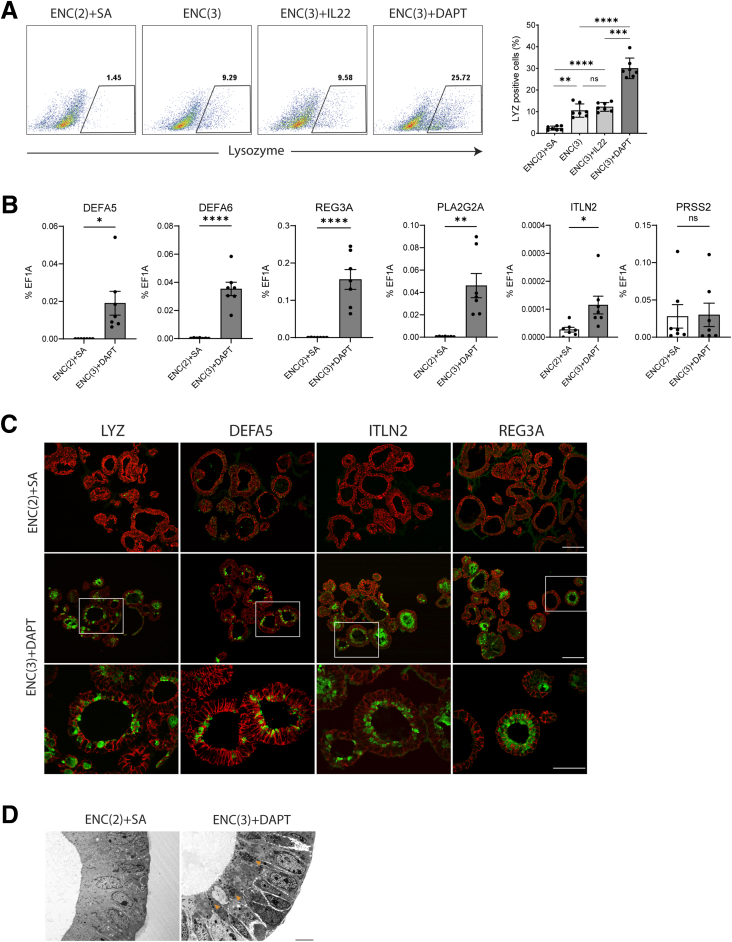
**Characterization of an enriched Paneth population in iPSC-derived eHIOs.** (*A*) Representative flow cytometry dot plots of lysozyme+ cells in eHIOs cultured in maintenance media (EGF [100 ng/mL], Noggin [100 ng/mL], and CHIR99021 [2 μM], SB202190 [10 μM], and A8301 [500 nM] (ENC(2)+SA) and those treated with an increased concentration of CHIR99021 [3 μM]; ENC(3)) either alone, or in combination with IL-22 (2 ng/mL; ENC(3) + IL22) or DAPT (10 μM; ENC(3)+D) with an accompanying graph showing data from 7 independent experiments. (*B*) qRT-PCR of Paneth cell-related genes in eHIOs cultured either in maintenance media or ENC(3)+D. Student’s *t*-test ∗*P* < .05; ∗∗∗*P* < .001; ∗∗∗∗*P* < .0001 as compared with maintenance media. Each value represents mean ± SEM. Data from 7 independent experiments. (*C*) Representative fluorescent images showing eHIOs, cultured either in maintenance media or ENC(3)+D, immunopositive for E-cadherin (*red*) and lysozyme, DEFA5, REG3A, and ITLN2 (*all green*). Scale bar is 100 μm for *upper 2 panels* and 25 μm for *lower panel*. *Lower panel* is enlarged images of *broken white rectangle* in *middle panel*. (*D*) Micrograph showing presence of electron-dense granules (*orange arrows*) in eHIOs treated with ENC(3)+D. Scale bar is 6 μm.

Having established the protocol to enrich the Paneth cell population in eHIOs, we then wished to confirm that these cells are biologically responsive to their milieu. Given that Paneth cells are at the interface between the mucosal immune system and microbiome, we wished to examine the responses to each. First, we examined the effects of IL-22 in the organoid modality for 5 days and found there was a significant increase in both the lysozyme+ and DEFA5+ population of cells (Figure 2*A*). Furthermore, although there were no changes in the expression of *DEFA5* and *DEFA6,* there were significant increases in the expression of *LYZ* and *PLA2G2A* (Figure 2*B*), thereby demonstrating their responsivity to cytokines.

**Figure 2:**
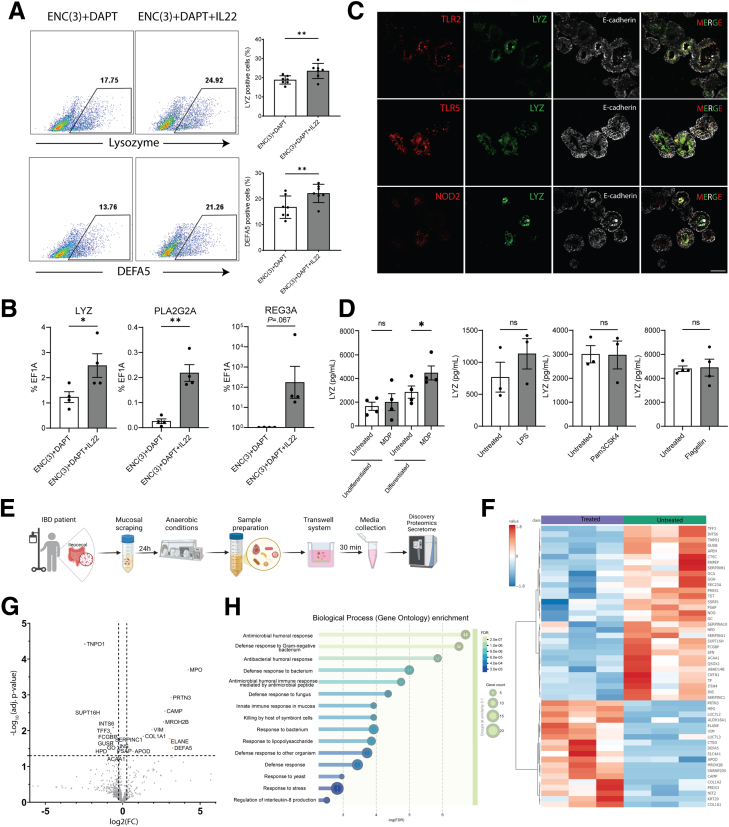
**Functional responses of iPSC-derived Paneth cells.** (*A*) Representative flow cytometry dot plots of lysozyme+ and DEFA5+ cells in eHIOs cultured with ENC(3)+D and treated either with/without IL22 (10 ng/mL) with an accompanying graph showing data from 7 independent experiments. (*B*) qRT-PCR of Paneth cell-related genes in ENC(3)+D-treated eHIOs with/without IL22 (10 ng/mL). Student’s *t*-test ∗*P* < .05; ∗∗*P* < .01 as compared with no addition of IL-22. Data from 4 independent experiments. (*C*) Representative fluorescent images showing enriched Paneth cell eHIOs are immunopositive for TLR2, TLR5, and NOD2 (*all red*), lysozyme (*green*), and E-cadherin (*white*). Scale bar is 50 μm. (*D*) Secretion of lysozyme in Paneth cell-enriched monolayers in response to apical administration of MDP, LPS, Pam3CSKJ4, and Flagellin. Undifferentiated monolayers were included as control (*right panel*). ∗∗∗*P* < .001 as compared with untreated control. Data from 3 to 4 independent experiments. (*E*) Schematic of studies utilizing microbial cultures obtained from patients with inflammatory bowel disease. (*F*) Heatmap of differentially secreted proteins in media obtained from Paneth cell-enriched monolayers collected following 30-minute exposure to bacterial enriched suspensions derived from Crohn’s disease mucosal scrapings. Samples are grouped as Treated (bacteria-exposed) or Untreated (control). Data represent mean values from 3 independent experiments. (*G*) Volcano plot of differential protein expression between Treated and Untreated secretome samples. Log_2_ fold change (log_2_FC) is plotted against –log_10_ adjusted *P* value (false discovery rate [FDR]). Proteins meeting significance thresholds (FDR < 0.05, |log_2_FC| > 1) are labeled. Data represent combined results from 3 independent experiments. (*H*) Gene Ontology (GO) Biological Process enrichment analysis of proteins significantly altered in the secretome following bacterial treatment. Terms are ranked by enrichment significance (–log_10_ FDR). Circle size reflects the number of associated proteins, and color indicates FDR. Analysis performed using STRING (version 12.0) based on data from 3 independent experiments.

As Paneth cell responses to microbial ligands/microbes are of considerable interest, we aimed to investigate such responses. We first investigated for the presence of various bacterial sensors in enriched Paneth cell eHIOs and confirmed the presence of TLR2, TLR5, and NOD2 (Figure 2*C*). As intestinal organoids are polarized towards the lumen, we dissociated enriched organoids and seeded them onto Transwells, whereby the luminal aspect could be accessed as previously described,^8^ so as to examine the effects of both microbial ligands and live bacteria and also quantify the secreted AMPs via enzyme-linked immunosorbent assay and mass spectrometry. We confirmed that Paneth cell markers remained highly enriched after seeding onto Transwells, indicating these cells were retained upon seeding (Supplementary Figure 2*C*) and also suggests the additional secretory cell types were present (Supplementary Figure 2*D*). We tested for ligands of the aforementioned receptors and found that the NOD2 ligand MDP caused a significant increase in the secretion of lysozyme (Figure 2*D*) but not by the Pam3CSK4, Flagellin, or LPS which is similar to a previous study.^9^ Importantly, MDP did not induce lysozyme secretion in undifferentiated eHIOs, indicating that this response is specific to the Paneth cell-enriched state. Finally, we co-cultured live bacteria, obtained from resected ileocolonic tissue (for schematic see Figure 2*E*), in Transwells with Paneth cell-enriched monolayers and found, via mass spectrometry, a significant increase in DEFA5 secretion, among others (Figure 2*F* and *G*), which additionally underscores a Paneth cell-specific response. To identify pathways altered by bacterial exposure, we performed Gene Ontology (GO) Biological Process enrichment analysis using STRING (version 12.0) in significantly changed secreted proteins (false discovery rate [FDR] <0.05). Consistent with the analysis, “Antimicrobial humoral response” and “Defense response to bacterium” were among the most upregulated pathways (Figure 2*H*).

Our goal was to enrich the Paneth cell population in eHIOs generated from iPSCs, and we demonstrate that we have achieved this aim by illustrating the presence of AMPs and their responses to various stimuli. Given that iPSCs can be generated from almost all individuals, this now permits a methodology whereby Paneth cells from a range of individuals, including those with Crohn’s disease, among others, can be generated and thus allows for studies into how genetic variations, microbial ligands/microbes, and components of the mucosal immune system influence this cell type.

## Supplementary Material

Supplementary Material

Extended PDF
