# Supplementary material for: A Method to Enrich Functional Human Paneth Cells in Induced Pluripotent Stem Cell-Derived Intestinal Organoids
Source: Cell Mol Gastroenterol Hepatol. 2026 Mar 18;20(7):101769. doi: 10.1016/j.jcmgh.2026.101769 (PMC13138201; doi:10.1016/j.jcmgh.2026.101769)
Supplement: Supplementary Material [file mmc1.pdf]

Supplementary data

Supplementary Figure 1

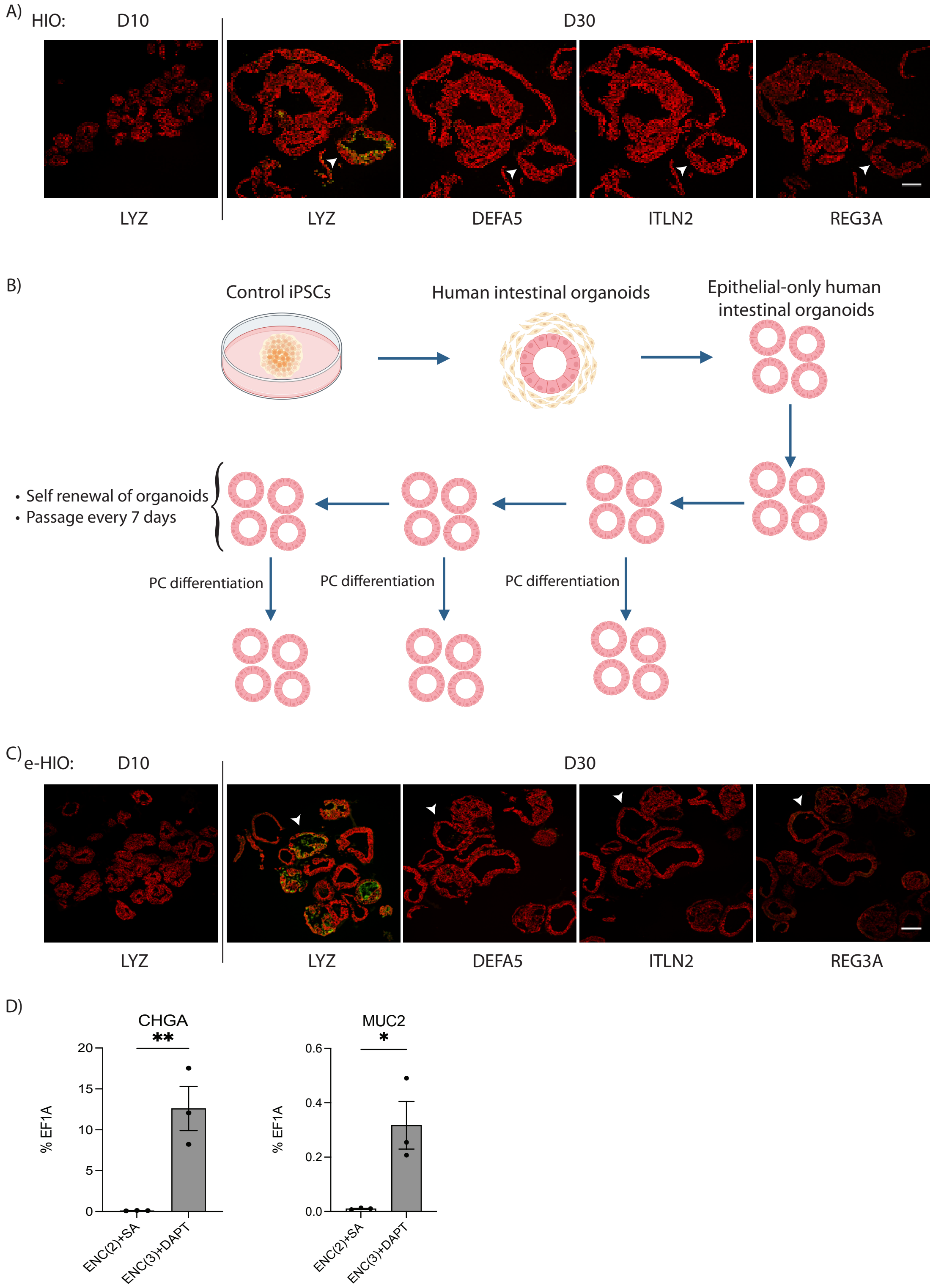

Supplementary Figure 1: Characterization of HIOs and eHIOs. (A) Representative fluorescent images of 10d and 30d HIOs immunopositive for E-cadherin (red) and lysozyme, DEFA5, REG3A and ITLN2 (green). Scale bar = 100μm. B) Schematic illustrating the generation of eHIOs from iPSCs. C) Representative fluorescent images of 10d and 30d e-HIOs immunopositive for E-cadherin (red) and lysozyme, DEFA5, REG3A and ITLN2 (green). Scale bar = 50μm. D) qPCR analysis of *CHGA* and *MUC2* expression in eHIOS cultured in ENC(2)+SA or ENC(3)+DAPT. Data represent mean ± SEM from 3 biological replicates. Students *t* test: **P* < .05 ***P* < .01 versus ENC(2)+SA.

Supplementary Figure 2.

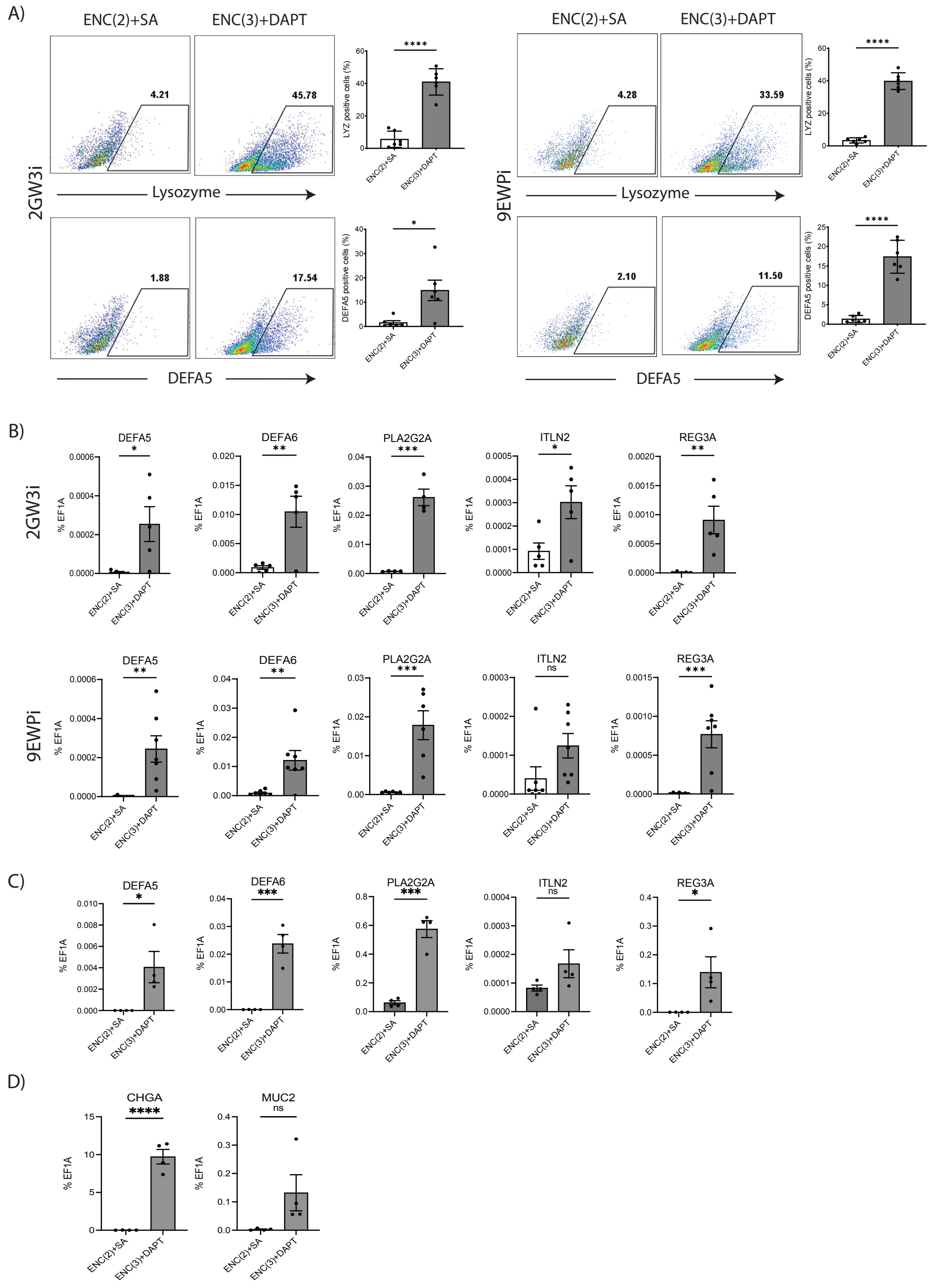

Supplementary Figure 2: Characterization of an enriched Paneth cell population in iPSC-derived eHIOs from 2 additional control lines, along with characterization of Paneth cell-enriched transwell cultures.

A) Representative flow cytometry dot plots showing lysozyme$^+$ and DEFA5$^+$ cells in eHIOs generated from control lines 2GW3i and 9EWPi, cultured in ENC(2)+SA or ENC(3)+DAPT media with an accompanying quantification graph from 6 independent experiments. B) qRT-PCR analysis of Paneth cell-related genes (*DEFA5*, *DEFA6*, *PLA2G2A*, *ITLN2*, and *REG3A*) in eHIOs from each control line cultured in ENC(2)+SA or ENC(3)+DAPT. (C) qRT-PCR characterization of Paneth cell markers **(***DEFA5*, *DEFA6*, *PLA2G2A*, *ITLN2*, *REG3A***)** in Paneth cell–enriched monolayers seeded on Transwell inserts and cultured in ENC(2)+SA or ENC(3)+DAPT. D) qRT-PCR analysis of enteroendocrine (*CHGA*) and goblet cell (*MUC2*) markers in Paneth cell-enriched transwell cultures maintained in ENC(2)+SA or ENC(3)+DAPT. Each value represents Mean $\pm$ SEM. Students *t* test *$P < .05$, **$P < .01$ and ***$P < .001$ as compared to ENC(2)+SA media.

Supplemental Table 1

Source/Specie	Proliferation media	Differentiation media	Time differentiating	Change in PC		Reference
				Mouse	Human	
Mouse small-intestinal crypts	EGF, Noggin, R-spondin1 (conditioned medium 5%final volume)	CHIR (2uM) and R-spondin1 (conditioned medium 10-20% final volume)	4 days	Immunostaining: increased LYZ$^+$; Number of PC/bud: increased	——	[1]
Mouse and human small-intestinal crypts	EGF, Noggin, R-spondin1, CHIR99021, Valproic acid	CHIR99021 (3uM) and DAPT (10uM)	4-7 days	Immunostaining: Day 4: 47% LYZ+ cells; Day 7: 85% LYZ+ cells; qPCR: ~15–20 fold Lyz1.	qPCR: ~3 fold Lyz	[2]
Human small-intestinal biopsies (upper and lower endoscopy)	50% conditioned media (Wnt3a, R-spondin, Noggin) + 50% fresh primary culture medium supplemented with Y-27632 and SB431542	5% or 50% of conditioned media (Wnt3a, R-spondin, Noggin) and DAPT (10uM)	2 -3 days	——	qPCR: 8–10 fold DEFA5 (ileal), 25–30 fold DEFA5 (duodenal).	[3]
Mouse small-intestinal crypts	EGF, Noggin, R-spondin1, CHIR99021, Valproic acid	CHIR (3uM) and DAPT (10uM)	2, 4 and 6 days	Immunostaining: 20–30% LYZ$^+$/DEFA$^+$ cells. qPCR: ~5–7 fold Lyz1, ~5–7 fold Mmp7, ~2.5–6 fold Defa1. Proteomics: upregulation of AMPs including DEFA (10), CRS peptides (5), ribonucleases (6), lectins (12), Lyz1, and Pla2g1b. Secretion: increased LYZ activity. Bacteria growth: suppresses growth of L. lactis (strain MG1363).	——	[4]
Mouse small-intestinal crypts	EGF, Noggin, R-spondin1	CHIR99021 (3uM) and DAPT (10uM)	2-7 days	RNA-seq / transcriptomics: Upregulation of Lyz1, Ang4, Reg3γ, Pla2g2a, and Defa2	——	[5]
Mouse small-intestinal crypts; adult human duodenum tissue	EGF, Noggin, R-spondin1, CHIR99021, Valproic acid	DAPT (10uM) and KPT-330	6 days	Immunostaining: increased LYZ$^+$ cells; Flow Cytometry: ~20% LYZ$^+$/CD24$^+$cells; LYZ Secretion: increased.	Immunostaining: increased LYZ$^+$ cells; LYZ Secretion: increased. scRNA-seq: increased LYZ expression.	[6]
Human small-intestinal crypts (ileum, duodenum)	EGF, Noggin, R-spondin, A-83-01, WNT surrogate (1:1000), CHIR99021	Removal of CHIR99021, WNT surrogate (1:30000), IL-22 (2ng/mL)	7 days	——	Immunostaining: 1–3% DEFA5+ cells. qPCR: increased DEFA5, DEFA6, REG3G. FACS: increased DEFA5+ cells. scRNA-seq: upregulation of DEFA6, PLA2G2A, PRSS2, REG3A, ITLN2, LYZ.	[7]
Mouse small-intestinal crypts; human terminal ileum	IntestiCult Organoid Growth Medium (StemCell Technologies, #06010)	IntestiCult Organoid Differentiation Medium (Stem Cell Techologies, #100-02140) + AS1842856 (1uM)	2 days	——	Immunostaining: DEFA5+ cells. qPCR: incresead DEFA5, DEFA6, REG3A, PLA2G2A, ITLN2.	[8]
Human small-intestinal biopsies	EGF, IGF-1, FGF-2, DMH1, Noggin, R-spondin, Gastrin I, A-83-01, CHIR99021, Trichostatin A (TSA), 2-phospho-L-ascorbic acid (pVc), CP673451	Removal of TSA and CP6773451 and addition of DAPT (10uM) and IL-22 (2ng/mL)	3 days	——	Immunostaining: 15% LYZ$^+$ and DEFA5 (not specified)	[9]

Supplemental Table 2

Media	Duration	Component	Final Concentration	Supplier / Catalog No.	Notes
**1. Organoid Medium (HIO Media)**<br>Base formulation used pre-MACS +A2:F14sort	Passaged every 7–10 days prior to MACS sort.<br>(Culture duration: 30 days)	Adv DMEM/F12	—	Gibco, 12634-010	Base medium
		B-27 Supplement	1×	Gibco, 17504-044	Added at all stages
		CHIR99021	2 μM	Tocris, 4423	Wnt activation; maintained through HIO culture
		Noggin	100 ng/mL	R&D Systems, 6057-NG	BMP inhibition
		EGF	100 ng/mL	R&D Systems, 236-EG	Supports epithelial growth
		L-glutamine	1% v/v	Gibco	Metabolic support
		Penicillin-Streptomycin	1% v/v	Gibco	Antimicrobial
**2. Epithelial-only Organoid Media (eHIO Media)**<br>Base formulation used *post-MACS sort*	Passaged every 7 days as part of cell culture maintenance.<br>Media refreshed every 2 days	HIO Media	—	—	Base formulation
		SB202190	10 μM	Tocris, 1264	p38 inhibitor; add fresh at each medium change
		A83-01	500 nM	Tocris, A83-01	TGF-β inhibitor; add fresh at each medium change
**3. Paneth Cell Enrichment Media (PC Media)**<br>Base formulation used for Paneth Cell differentiaion	**1.** Culture single epithelial cells in matrigel domes for 2 days in eHIO media; **2.** On 3rd day switch to PC media for 5 days. **3.** Split developed organoid structure into new matrigel domes from1 to 2 parts and culture in PC media for another 5 days (no single cell dissociation); Media refreshed every 2 days.<br>(Culture duration: 12days; 2 days eHIO media +10 days PC Media)	HIO Media (as above)	—	—	Base formulation
		CHIR99021	3 μM	Tocris, 4423	Wnt activator; add fresh at each medium change
		DAPT	10 μM	R&D Systems, 2634/10	Notch inhibitor; add fresh at each medium change; drives Paneth cell differentiation

Supplemental Table 2. Media formulations used for HIO and eHIO culture and Paneth cell enrichment. This table lists all components, final concentrations, suppliers, and catalog numbers for each media condition used in the study, including HIO maintenance media, epithelial-only HIO (eHIO) expansion media, and the modified ENC(3) and ENC(3)+DAPT formulations. Stage-specific notes are provided to enable full replication of the differentiation and enrichment protocol

Methods:

Statement of Ethics

All the cell lines and protocols in the present study were carried out in accordance with the guidelines approved by the stem cell research oversight committee and institutional review board at the Cedars-Sinai Medical Center under the auspice of the institutional review board stem cell research oversight committee protocols Pro00027264 and Pro0004857. All authors had access to the study data and had reviewed and approved the final manuscript.

Cell Lines and Culture Conditions

The CS03iCTR-n1, CS2GW3iCTR-n1, and CS9EWPiCTR-n1 iPSC lines were obtained from the iPSC Core at Cedars-Sinai Medical Center (Los Angeles, CA, USA) and were derived from healthy control subjects. These lines were fully characterized for pluripotency markers and confirmed to be karyotypically normal. iPSCs were maintained in an undifferentiated state on Matrigel®-coated plates (Corning, 354234; Corning, NY, USA) in mTeSR™1 medium (STEMCELL Technologies, 85851; Vancouver, BC, Canada) under feeder-free conditions. Cells were passaged every 4–5 days using ReLeSR™ (STEMCELL Technologies, 100-0483; Vancouver, BC, Canada).

HIO Generation from iPSCs

The generation of human intestinal organoids (HIOs) from iPSCs was performed using a multistep protocol as previously described [10]. Briefly, iPSCs were directed to definitive endoderm, followed by hindgut structures, and ultimately into organoids. All HIOs were cultured in medium containing CHIR99021 (2 µM; Tocris Bioscience, 4423; Bristol, UK), Noggin (100 ng/mL; R&D

Systems, 6057-NG; Minneapolis, MN, USA), EGF (100 ng/mL; R&D Systems, 236-EG; Minneapolis, MN, USA), B-27 Supplement (1×; Gibco, 17504-044; Waltham, MA, USA), and DMEM/F12 (Gibco, 12634-010; Waltham, MA, USA), supplemented with penicillin/streptomycin and L-glutamine (5% v/v). All organoid cultures were embedded in standard Matrigel® Basement Membrane Matrix (Corning 354234), a non–growth factor–reduced formulation used consistently across all experiments. Organoids were passaged every 7–10 days and subjected to magnetic-activated cell sorting (MACS) after 30 days of differentiation.

Generation of Purified Epithelial Cell Cultures

HIOs were removed from Matrigel and washed 3 times in Dulbecco's Phosphate-Buffered Saline (DPBS; Gibco, 14190-144; Waltham, MA, USA) and subsequently incubated in TrypLE™ Select (Gibco, 12563-029; Waltham, MA, USA) for 12 minutes until the organoids were completely dissociated to a single-cell suspension. Dissociated cells were incubated with EpCAM MicroBeads (Miltenyi Biotec, 130-061-101; Bergisch Gladbach, Germany) for 30 minutes at 4 °C. EpCAM$^+$ cells (eHIOs) were obtained via the autoMACS® Pro Separator (Miltenyi Biotec, Bergisch Gladbach, Germany). Following isolation, EpCAM$^+$ epithelial cells were resuspended in Matrigel as single cells, where they self-organized and expanded to form epithelial-only HIOs (eHIOs). eHIOs were maintained in organoid medium supplemented with SB202190 (10 µM; Tocris Bioscience, 1264; Bristol, UK) and A83-01 (500 nM; Tocris Bioscience, A83-01; Bristol, UK).

Enrichment of Paneth Cells

eHIOs were split once a week, and the medium was refreshed every 3 days. For passaging, eHIOs were removed from Matrigel, washed 3 times with DPBS (Gibco, 14190-144; Waltham, MA, USA), and incubated in TrypLE™ Select (Gibco, 12563-029; Waltham, MA, USA) for 12 minutes until fully dissociated into a single-cell suspension. Cells were then seeded into fresh Matrigel domes and maintained in eHIO expansion medium for 2 days. After 2 days, the culture medium was replaced with differentiation medium containing increased CHIR99021 (3 µM; Tocris Bioscience, 4423; Bristol, UK), IL-22 (2ng/mL; R&D Systems, 782IL; Minneapolis, MN, USA) and DAPT (10 µM; R&D Systems, 2634/10; Minneapolis, MN, USA) to promote Paneth cell differentiation. Organoids were maintained in this differentiation medium for 5 days. Subsequently, the expanded eHIOs were replated without enzymatic dissociation and cultured for an additional 5 days in the same differentiation medium. On Day 12 of the differentiation protocol, cells were harvested and analyzed for Paneth cell enrichment. To assess Paneth cell antimicrobial function at the transcriptional level, eHIOs were stimulated with IL-22 (10 ng/mL; R&D Systems, 782IL; Minneapolis, MN, USA) for 72 or 120 hours, starting on days 10 and 12 of the differentiation protocol, respectively.

Quantitative Polymerase Chain Reaction (qPCR)

RNA was extracted from eHIOs using the RNeasy® Mini Kit (Qiagen, 74106; Hilden, Germany). Complementary DNA (cDNA) was synthesized from 500 ng of RNA using the High-Capacity cDNA Reverse Transcription Kit (Applied Biosystems™, 4368814; Foster City, CA, USA). Quantitative real-time PCR was performed using SYBR™ Select Master Mix (Applied Biosystems™, 4472908;

Foster City, CA, USA) on a Bio-Rad CFX384 Real-Time System (Bio-Rad Laboratories, Hercules, CA, USA). Primer sequences are listed in

Table 1.

Gene name	Forward Primer	Reverse Primer
EF1A	TATTGGATTGCCACACGGCT	AAAGCGACCCAAAGGTGGAT
LYZ	TTTCTGTTACGGTCCAGGGC	ACACATCCAGTTTGCTAGGCT
DEFA5	TGGGGAAGACAACCAGGAC	TTCGGCAATAGCAGGTGG
DEFA6	GAGGATGCAAGCTCAAGTCTTAG	TGCAATGGCAAGTGAAAGC
REG3A/G	ACTGCTATGCCTTGTTTTTGTC	CTACTCCACTCCCAACCTTCTC
SPLA2G2A	CGCACTCAGTTATGGCTTCTAC	GTGATTCTGCTCCCCGAG
ITLN2	CTCCTTTTCTTCCCTGCCTAG	TCTGGTAGACAACACCATTCTTG
PRSS2	CCCTCATCAGCGAACAGTG	CAGGATGTCATTGTCCAGAGTC
TLR2	CGGCGTTCTCTCAGGTGA	TGGCATTGTCCAGTGCTTC
TLR5	TGCTACTGACAACGTGGCTTC	ACATCTGAGGCTCCGACATC
NOD2	GTGGAGAACATGCTGGACCT	GCCAATGGGACTGGTAATTC

Immunocytochemistry and Microscopy

eHIOs were fixed in 4% paraformaldehyde (Electron Microscopy Sciences, 15714-5; Hatfield, PA, USA), transferred to 30% sucrose overnight at 4 °C, embedded in Tissue-Tek® O.C.T. Compound (Sakura Finetek, 4583; Torrance, CA, USA), and cryosectioned at 8 µm thickness. Sections were blocked with 10% normal donkey serum in 0.5% Triton X-100 and incubated overnight at 4 °C with primary antibodies (Table 2). The following day, sections were rinsed and incubated with Alexa Fluor 488 (1:1000; Invitrogen, A21202; Carlsbad, CA, USA), Alexa Fluor 594 (1:1000; Invitrogen, A32758; Carlsbad, CA, USA) or Alexa Fluor 647 (1:1000; Invitrogen, A21447; Carlsbad, CA, USA) for 1 hour at room temperature, followed by DAPI counterstaining (1:5000; Invitrogen, H3570; Carlsbad, CA, USA). Slides were imaging using Nikon Eclipse Ti- A1R HD.

Antigen	Host Species	Manufacturer	Catalog number	Dilution
Lysozyme	Mouse	Novus Biologicals	NB100-63062	1/500
Lysozyme	Rabbit	Invitrogen	PA5-16668	1/250
NOD2	Mouse	Novus Biologicals	NB100-524	1/100
DEFA5	Mouse	Novus Biologicals	NB110-60002	1/1250
ITLN2	Mouse	R&D Systems	MAB8004	1/100
Reg3A	Mouse	R&D Systems	MAB5965	1/200
E-cadherin	Goat	R&D Systems	AF648	1/1000
TLR2	Rabbit	Novus Biologicals	NB100-56720	1/250
TLR5	Mouse	Novus Biologicals	NBP2-24787	1/250

Organoid Epithelial Cell Monolayer

Paneth cell-enriched eHIOs were dissociated into single cells using TrypLE™ Select (Gibco, 12563-029; Waltham, MA, USA) and seeded onto 6.5 mm Transwell® inserts (STEMCELL Technologies, 38024; Vancouver, BC, Canada) pre-coated with Matrigel® (Corning, 354234; Corning, NY, USA) at a density of 4.0×10^5 cells per well. Cells were cultured in differentiation medium for 24 hours to allow monolayer formation and stabilization prior to stimulation.

Enzyme-Linked Immunosorbent Assay (ELISA)

Media from transwell inserts were collected after exposure to LPS (100 µg/mL; Sigma-Aldrich, L2880; St. Louis, MO, USA), Pam3CSK4 (10 µg/mL; Novus Biologicals, NBP2-25297; Centennial, CO, USA), and MDP (100 µg/mL; InvivoGen, tlrl-mdp; San Diego, CA, USA) for 30 minutes. Human lysozyme ELISA (Novus Biologicals, NBP2-60511; Centennial, CO, USA) was performed according to the manufacturer's instructions. Absorbance at 450 nm and 570 nm was measured using a SpectraMax M3 microplate reader (Molecular Devices, San Jose, CA, USA).

Flow Cytometry

eHIOs were removed from Matrigel, washed three times with DPBS (Gibco, 14190-144; Waltham, MA, USA), and incubated in TrypLE™ Select (Gibco, 12563-029; Waltham, MA, USA) for 12 minutes at 37 °C to generate a single-cell suspension. Dissociated cells were passed through a 70 µm mini cell strainer (pluriSelect, 43-10070-40; Leipzig, Germany) to remove aggregates and debris. Cells were then fixed in Flow Cytometry Fixation Buffer (R&D Systems, FC004; Minneapolis, MN, USA) and permeabilized using Flow Cytometry Permeabilization/Wash Buffer (R&D Systems, FC005; Minneapolis, MN, USA), following the manufacturer's protocol. Permeabilized cells were incubated with unconjugated primary antibodies: anti-Lysozyme (1:100; Novus Biologicals, NB100-63062; Centennial, CO, USA) and anti-DEFA5 (1:2000; Novus Biologicals, NB110-60002; Centennial, CO, USA) for 30 minutes at 4 °C. After three washes, cells were incubated with Alexa Fluor 488-conjugated secondary antibody (1:100; Invitrogen, A21202; Carlsbad, CA, USA) for 30 minutes at 4 °C in the dark. Samples were analyzed using a BD LSR Fortessa™ Cell Analyzer (BD Biosciences, San Jose, CA, USA), and data were analyzed with FlowJo software (BD Biosciences, San Jose, CA, USA).

Transmission Electron Microscopy

Organoid samples were fixed in 2.5% glutaraldehyde and 2% formaldehyde in 0.1 M sodium cacodylate at 4 ˚C. After fixation, samples were dehydrated and embedded in Epon 812 (Polysciences). The ultrathin sections were stained with 1% uranyl acetate and 3% lead citrate. Ultimately, the images were taken using HT7700 transmission electron microscope (HITACHI) at 1,200x direct magnification.

Bacterial Isolation from Crohn's Disease Intestinal Resections

Intestinal tissue specimens were collected aseptically from patients with Crohn's disease undergoing ileocolonic resections. Immediately following surgical excision, tissues were placed in sterile containers and transferred from the operating room to a biosafety cabinet for processing within 1 h of resection. Tissues were rinsed with sterile DPBS (Gibco, 14190-144; Waltham, MA, USA) until all visible surface blood was removed. Sutured ends were excised, and the bowel was opened longitudinally. Mucosal scrapings were obtained, yielding 200–500 µL of mucosal material per specimen, which was transferred into sterile microcentrifuge tubes. Mucosal scrapings were immediately placed in an anaerobic chamber (Coy Laboratory Products, 602000; Grass Lake, MI, USA) maintained at 90% N_2, 5% CO_2, and 5% H_2. Samples were homogenized in 1 mL of reduced sterile DPBS and an aliquot (100 µL) of the homogenate was inoculated into pre-reduced 9.9 mL brain heart infusion (BHI; Sigma-Aldrich, B21202; St. Louis, MO, USA) and 9.9 mL Gifu Anaerobic Medium (GAM; HiMedia, M189; Mumbai, India). Cultures were incubated anaerobically at 37 °C for ≥12 h. Overnight cultures from both media were pooled, and bacterial suspensions were normalized to an optical density at 600 nm (OD_{600}). TW was treated with the pooled bacterial suspension at a final concentration of OD_{600} = 0.5 for 30 min, after which the media was collected for analysis.

Proteomics Sample Digest

Samples were digested using the SP3 protocol. Briefly, 20 µg of protein was brought to 60 µL with lysis buffer containing 6 M urea, 1 M ammonium bicarbonate, and 5% SDS (Thermo Fisher, 20230; Waltham, MA, USA). Proteins were reduced with 16.8 µL of 200 mM dithiothreitol (DTT;

Sigma-Aldrich, D9779; St. Louis, MO, USA) for 30 min at 37 °C with shaking at 300 rpm, then alkylated with 21.2 µL of 400 mM iodoacetamide (IAA; Sigma-Aldrich, I1149; St. Louis, MO, USA) at room temperature for 30 min in the dark. The volume was brought to 160 µL with Tris-HCl pH 8 (Thermo Fisher, AM9856; Waltham, MA, USA), and 5 µL of bead suspension (10:1 mass ratio of beads to protein; 1:1 mixture of hydrophilic/hydrophobic beads; Cytiva, 29152179; Marlborough, MA, USA) was added and vortexed. Samples were adjusted to 70% acetonitrile (ACN; Sigma-Aldrich, 34851; St. Louis, MO, USA) v/v and incubated for 18 min. Solvent was removed on-magnet, and samples were washed with 2× 80% ethanol (EtOH; Sigma-Aldrich, 459844; St. Louis, MO, USA) and 2× ACN (200 µL each). After complete solvent removal, samples were resuspended in 50 mM Tris-HCl pH 8 with 10 mM $CaCl_2$ (Sigma-Aldrich, C1016; St. Louis, MO, USA) and digested with trypsin (1:20 enzyme:protein; Promega, V5111; Madison, WI, USA). Samples were bath-sonicated for 5 min, then incubated for 18 h at 37 °C with shaking at 1200 rpm. After digestion, samples were removed from beads and brought to 0.1% formic acid (FA; Sigma-Aldrich, F8775; St. Louis, MO, USA) and 2% DMSO (Sigma-Aldrich, 276855; St. Louis, MO, USA) for LC-MS/MS injection.

LC-MS/MS Analysis

Approximately 500 ng of peptides from digested samples were analyzed on a Thermo Orbitrap Astral mass spectrometer (Thermo Fisher, A12345; Waltham, MA, USA) coupled to a NeoVanquish LC (Thermo Fisher, NLC123; Waltham, MA, USA). A blank injection was included after every three samples to assess carryover. Peptides were separated using a 24 min gradient with solvent A (0.1% formic acid in water; Sigma-Aldrich, F8875; St. Louis, MO, USA) and solvent

B (80% acetonitrile, 0.1% formic acid; Sigma-Aldrich, 34851; St. Louis, MO, USA) at 1.2 µL/min. The gradient was: 0 min, 4% B; 2 min, 9% B; 13 min, 25% B; 17 min, 35% B. LC was performed in direct injection mode using a PepSep C18 column (150 µm ID × 15 cm, 1.5 µm; Bruker, 100367; Billerica, MA, USA) coupled to a nano source (Thermo EasySpray, ES081; Waltham, MA, USA). MS data were acquired in data-independent acquisition (DIA) mode from 380–980 Da with 240k Orbitrap resolution, 10 ms maximum injection time for MS1, and 4 ms maximum injection time for MS2 with 4 Th windows.

Data Analysis

MS raw data files were searched against UniProt human reviewed protein sequence entries (accessed April 2023) using DIA-NN (v 1.8.1) [11] in library free mode with default parameters. Based on recent comparisons with library-based approaches, DIA-NN in library-free mode has been found to produce results that are comparable or better than those of experimental library-based searches while being freely available and was hence chosen for the analysis of all data (PMID: 36609502). The output protein group matrix from DIA-NN was used to perform downstream analysis using MetaboAnalyst 6.0 [12].

Gene ontology enrichment analysis

Proteins identified in the secretome were filtered for statistical significance using a p-value < 0.05 and false discovery rate (FDR) < 0.05. The filtered list of proteins was submitted to STRING (version 12.0; https://string-db.org) for functional enrichment analysis of Gene Ontology (GO) Biological Processes, using *Homo sapiens* as the reference species. STRING calculates enrichment

using a hypergeometric test and corrects for multiple comparisons with the Benjamini–Hochberg procedure. Enrichment results are reported as STRING-derived FDR values.

Statistical Analysis

All data are presented as mean ± standard error of the mean (SEM). Statistical analyses were performed using GraphPad Prism version 9.2.0 (GraphPad Software, San Diego, CA, USA). Comparisons among multiple groups were conducted using two-way analysis of variance (two-way ANOVA) followed by appropriate post hoc tests. Unpaired or paired t-tests were used for comparisons between two groups, as appropriate. A p-value $< .05$ was considered statistically significant.

Supplementary References:

[1] Farin HF, Van Es JH, Clevers H. Redundant sources of Wnt regulate intestinal stem cells and promote formation of Paneth cells. Gastroenterology 2012;143(6):1518-29 e7.

[2] Yin X, Farin HF, van Es JH, Clevers H, Langer R, Karp JM. Niche-independent high-purity cultures of Lgr5+ intestinal stem cells and their progeny. Nat Methods 2014;11(1):106-12.

[3] VanDussen KL, Marinshaw JM, Shaikh N, Miyoshi H, Moon C, Tarr PI, Ciorba MA, Stappenbeck TS. Development of an enhanced human gastrointestinal epithelial culture system to facilitate patient-based assays. Gut 2015;64(6):911-20.

[4] Mead BE, Ordovas-Montanes J, Braun AP, Levy LE, Bhargava P, Szucs MJ, Ammendolia DA, MacMullan MA, Yin X, Hughes TK, Wadsworth MH, 2nd, Ahmad R, Rakoff-Nahoum S, Carr SA, Langer R, Collins JJ, Shalek AK, Karp JM. Harnessing single-cell genomics to improve the physiological fidelity of organoid-derived cell types. BMC Biol 2018;16(1):62.

[5] Treveil A, Sudhakar P, Matthews ZJ, Wrzesinski T, Jones EJ, Brooks J, Olbei M, Hautefort I, Hall LJ, Carding SR, Mayer U, Powell PP, Wileman T, Di Palma F, Haerty W, Korcsmaros T. Regulatory network analysis of Paneth cell and goblet cell enriched gut organoids using transcriptomics approaches. Mol Omics 2020;16(1):39-58.

[6] Mead BE, Hattori K, Levy L, Imada S, Goto N, Vukovic M, Sze D, Kummerlowe C, Matute JD, Duan J, Langer R, Blumberg RS, Ordovas-Montanes J, Yilmaz OH, Karp JM, Shalek AK. Screening for modulators of the cellular composition of gut epithelia via organoid models of intestinal stem cell differentiation. Nat Biomed Eng 2022;6(4):476-94.

[7] He GW, Lin L, DeMartino J, Zheng X, Staliarova N, Dayton T, Begthel H, van de Wetering WJ, Bodewes E, van Zon J, Tans S, Lopez-Iglesias C, Peters PJ, Wu W, Kotlarz D, Klein C, Margaritis T, Holstege F, Clevers H. Optimized human intestinal organoid model reveals interleukin-22-dependency of paneth cell formation. Cell Stem Cell 2022;29(9):1333-45 e6.

[8] Eng SJ, Nonnecke EB, de Lorimier AJ, Ali MR, Tsolis RM, Bevins CL, Ashwood P. FOXO inhibition rescues alpha-defensin expression in human intestinal organoids. Proc Natl Acad Sci U S A 2023;120(47):e2312453120.

[9] Yang L, Wang X, Zhou X, Chen H, Song S, Deng L, Yao Y, Yin X. A tunable human intestinal organoid system achieves controlled balance between self-renewal and differentiation. Nat Commun 2025;16(1):315.

[10] Workman MJ, Gleeson JP, Troisi EJ, Estrada HQ, Kerns SJ, Hinojosa CD, Hamilton GA, Targan SR, Svendsen CN, Barrett RJ. Enhanced Utilization of Induced Pluripotent Stem Cell-Derived Human Intestinal Organoids Using Microengineered Chips. Cell Mol Gastroenterol Hepatol 2018;5(4):669-77 e2.

[11] Demichev V, Messner CB, Vernardis SI, Lilley KS, Ralser M. DIA-NN: neural networks and interference correction enable deep proteome coverage in high throughput. Nat Methods 2020;17(1):41-4.

[12] Pang Z, Lu Y, Zhou G, Hui F, Xu L, Viau C, Spigelman AF, MacDonald PE, Wishart DS, Li S, Xia J. MetaboAnalyst 6.0: towards a unified platform for metabolomics data processing, analysis and interpretation. Nucleic Acids Res 2024;52(W1):W398-W406.
