# Supplementary material for: A Method to Enrich Functional Human Paneth Cells in Induced Pluripotent Stem Cell-Derived Intestinal Organoids
Source: Cell Mol Gastroenterol Hepatol. 2026 Mar 18;20(7):101769. doi: 10.1016/j.jcmgh.2026.101769 (PMC13138201; doi:10.1016/j.jcmgh.2026.101769)
Supplement: Extended PDF [file mmc2.pdf]

cmgh
CELLULAR AND MOLECULAR GASTROENTEROLOGY AND HEPATOLOGY

TECHNICAL ADVANCES

A Method to Enrich Functional Human Paneth Cells in Induced Pluripotent Stem Cell-Derived Intestinal Organoids

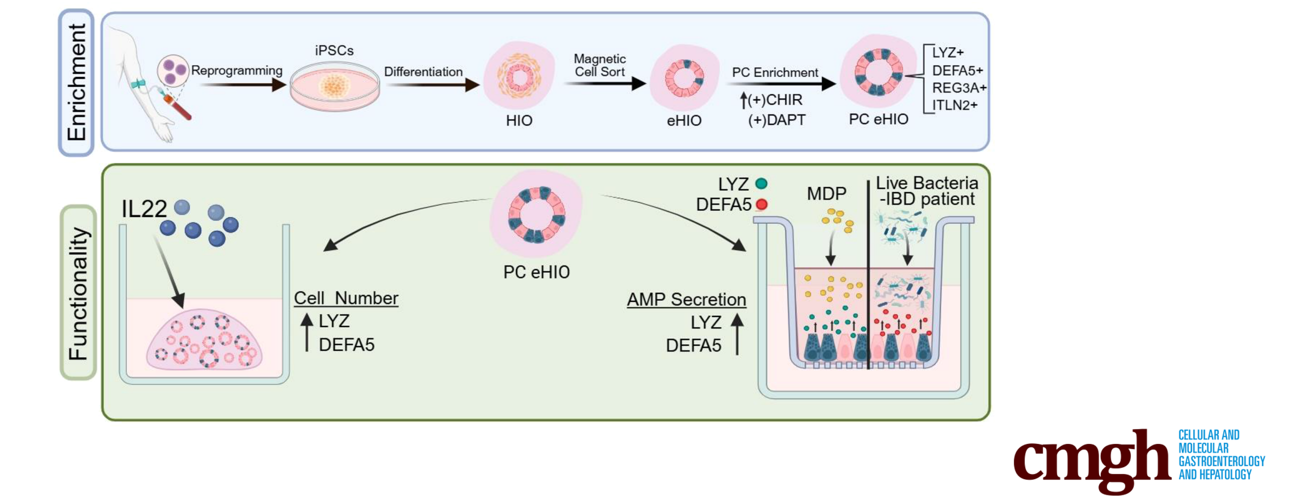

Paneth cells are a specialized intestinal epithelial subtype that reside in the crypt of the small intestine, and whose major function is to produce antimicrobial peptides (AMPs). Genetic variants in the *NOD2* and *ATG16L1* genes[1,2] are associated with an altered Paneth cell phenotype but the confounding influences of the microbiome and mucosal immune system make it challenging to determine whether genetic variations or environmental influences are the cause of these altered phenotypes. Therefore, our goal was to develop a biologically responsive in vitro Paneth cell model that would permit an examination of both intrinsic and extrinsic influences in a controlled reductionist system.

We chose to utilize induced pluripotent stem cells (iPSCs) for the source of our human intestinal organoids (HIOs), as this is a donor cell type that can be obtained either from a simple blood draw[3] or lymphoblastoid cell lines from numerous-well characterized biorepositories,[4] and iPSC-derived HIOs have previously been shown to be representative of the small intestine.[5] We directed a control iPSC line (CS03iCTR-n1) to HIOs as previously described,[6] and the initial characterization of our 10d iPSC-derived HIOs revealed a complete absence of lysozyme+ cells. After 30 days, only 2 organoids in one series of experiments were found to be lysozyme+ but were negative for additional AMPs (Supplementary Figure 1*A*). Given the near absence of Paneth cells, we thus aimed to enrich our organoids for this cell type. Numerous attempts have been made to enrich for Paneth cells in various modalities such as murine organoids and human biopsy-derived intestinal organoids (Supplementary Table 1), and so to test a subset of these approaches, we first purified the epithelial cellular component of 10-day and 30-day HIOs, cultured them as epithelial-only HIOs (eHIOs), which could be serially passaged every 7 days, with the goal of examining such approaches (Supplementary Figure 1*B*). Given that we found that eHIOs from 10-day iPSC-derived HIOs again had no lysozyme+ cells, whereas those from 30-day iPSC-derived HIOs contained a small presence (Supplementary Figure 1*C*), we thus utilized eHIOs from the 30-day timepoint in all studies listed below.

Previous attempts to enrich for Paneth cells in the organoid modeling system generally utilized the Notch inhibitor, DAPT, and the GSK-inhibitor CHIR99021, and although this resulted in increased numbers of lysozyme+ cells (∼ 20-85%) in murine intestinal organoids, human biopsy-derived organoids were generally limited to increases in the expression of Paneth cell-related genes (Supplementary Table 1).Two studies attempted to enrich via interleukin (IL)-22 addition in biopsy-derived human intestinal organoids and this resulted in the presence of ∼1% to 15% Paneth cells (Supplementary Table 1). To assess both of these approaches in our iPSC-derived cells, we first modified our eHIO maintenance media and found the removal of the small molecules SB202190/A83-01 and increased concentration of CHIR99021 ($2\mu M \rightarrow 3\mu M$) (Methods and Supplementary Table 2) led to a significant increase in lysozyme+ cells from ∼2.5% to ∼10%, and although there was no significant increase with the addition of IL-22, there was a significant increase to ∼30% lysozyme+ cells with the addition of DAPT (Figure 1*A*). We then assessed for previously identified human Paneth cell-related genes[7] and found that increased CHIR99021 and DAPT led to a significant increase in the expression of *DEFA5, DEFA6, PLA2G2A, REG3A*, and *ITLN2* mRNA (Figure 1*B*) as compared with those in maintenance media. We then confirmed via immunofluorescent stainings that these

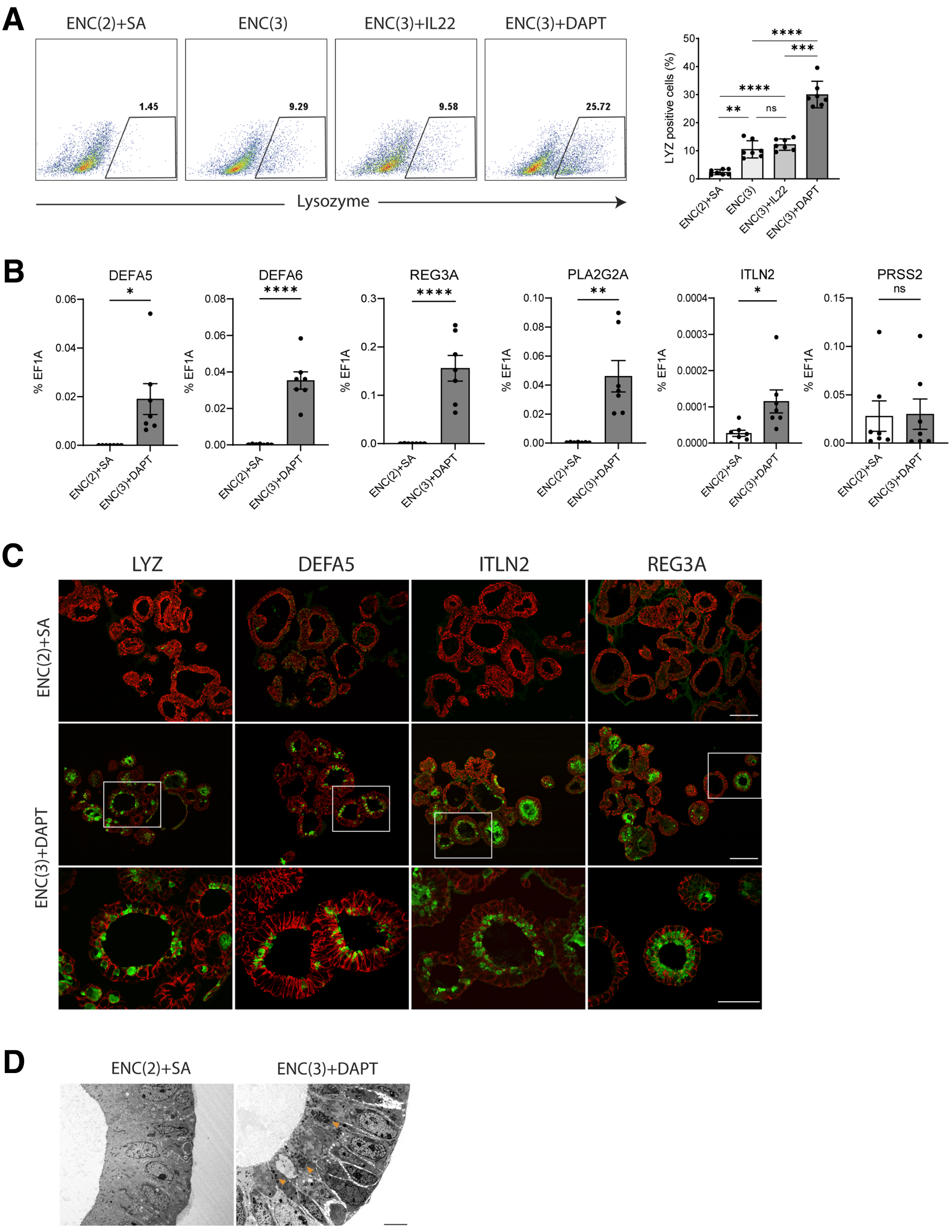
A
ENC(2)+SA
ENC(3)
ENC(3)+IL22
ENC(3)+DAPT
1.45
9.29
9.58
25.72
Lysozyme
LYZ positive cells (%)
B
DEFA5
DEFA6
REG3A
PLA2G2A
ITLN2
PRSS2
% EF1A
C
LYZ
DEFA5
ITLN2
REG3A
ENC(2)+SA
ENC(3)+DAPT
D
ENC(2)+SA
ENC(3)+DAPT

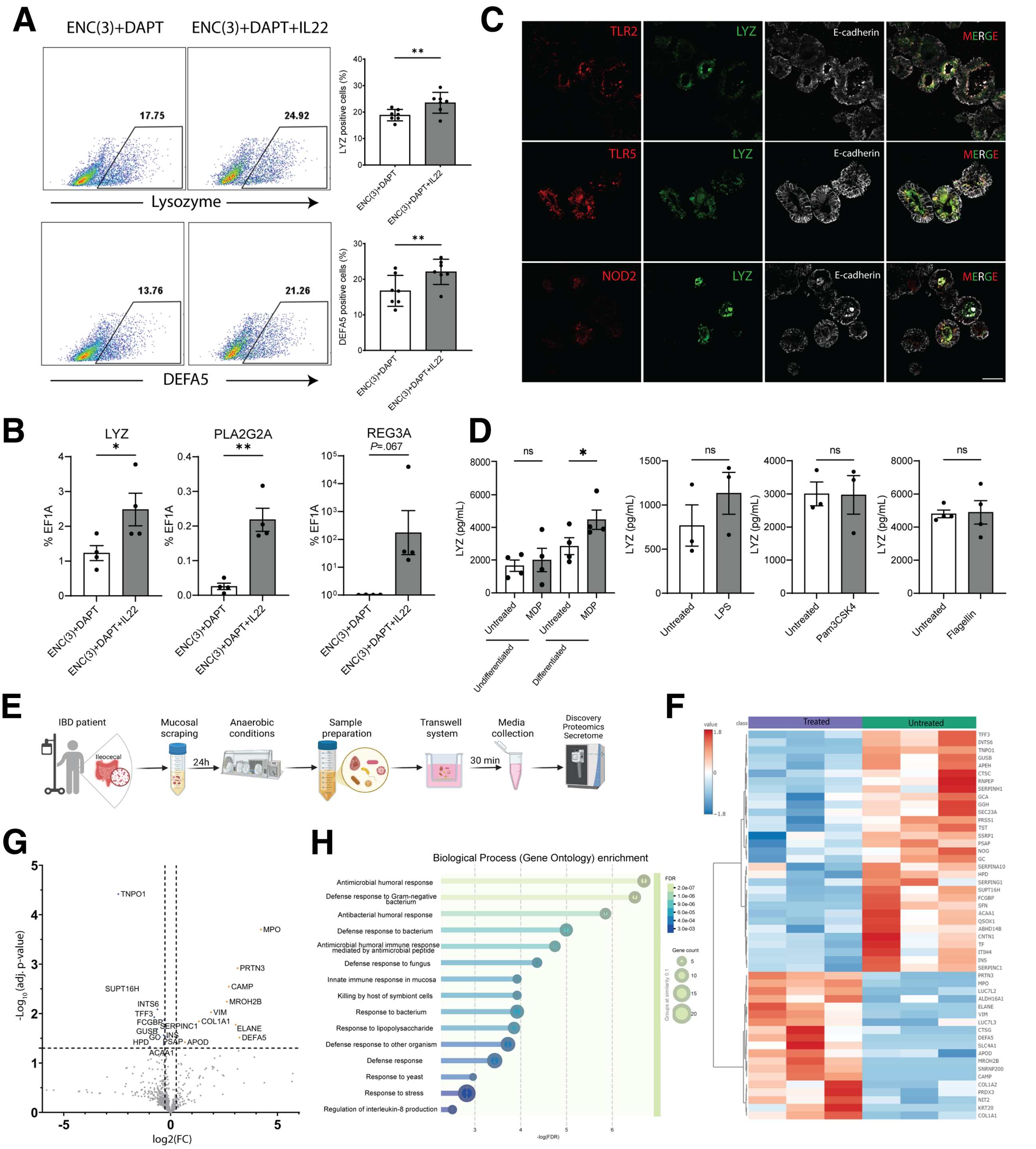

Figure 1. (*See previous page*). **Characterization of an enriched Paneth population in iPSC-derived eHIOs.** (*A*) Representative flow cytometry dot plots of lysozyme+ cells in eHIOs cultured in maintenance media (EGF [100 ng/mL], Noggin [100 ng/mL], and CHIR99021 [2 μM], SB202190 [10 μM], and A8301 [500 nM] (ENC(2)+SA) and those treated with an increased concentration of CHIR99021 [3 μM]; ENC(3)) either alone, or in combination with IL-22 (2 ng/mL; ENC(3) + IL22) or DAPT (10 μM; ENC(3)+D) with an accompanying graph showing data from 7 independent experiments. (*B*) qRT-PCR of Paneth cell-related genes in eHIOs cultured either in maintenance media or ENC(3)+D. Student's *t*-test **P* < .05; ****P* < .001; *****P* < .0001 as compared with maintenance media. Each value represents mean ± SEM. Data from 7 independent experiments. (*C*) Representative fluorescent images showing eHIOs, cultured either in maintenance media or ENC(3)+D, immunopositive for E-cadherin (*red*) and lysozyme, DEFA5, REG3A, and ITLN2 (*all green*). Scale bar is 100 μm for *upper 2 panels* and 25 μm for *lower panel*. *Lower panel* is enlarged images of *broken white rectangle* in *middle panel*. (*D*) Micrograph showing presence of electron-dense granules (*orange arrows*) in eHIOs treated with ENC(3)+D. Scale bar is 6 μm.

factors led to numerous cells within eHIOs to possess not only granulated lysozyme, but also granulated DEFA5, REG3A, and ITLN2 (Figure 1*C*), and transmission electron microscopy revealed the presence of electron dense granules (Figure 1*D*), all of which suggests the presence of an enriched Paneth cell population. Given that Notch inhibition can globally influence secretory differentiation, we also observed increased expression of both *CHGA* and *MUC2*, which suggests that both enteroendocrine and goblet cells are obtained also (Supplementary Figure 2*C*). Finally, to confirm that this protocol is not restricted to the aforementioned control iPSC line, we demonstrated that the addition of increased CHIR99021 and DAPT to eHIOs, generated from 2 other control iPSC lines, also gave enriched Paneth cell populations. Under ENC(3)+DAPT conditions, CS2GW3i eHIOs contained 45.8% LYZ^+ and 17.5% $DEFA5^+$ cells, whereas CS9EWPi eHIOs showed 33.6% LYZ^+ and 11.5% $DEFA5^+$ cells (Supplementary Figure 2*A*), and there was significantly upregulated *DEFA5*, *DEFA6*, *PLA2G2A*, and *REG3A* in both lines (Supplementary Figure 2*B*).

Having established the protocol to enrich the Paneth cell population in eHIOs, we then wished to confirm that these cells are biologically responsive to their milieu. Given that Paneth cells are at the interface between the mucosal immune system and microbiome, we wished to examine the responses to each. First, we examined the effects of IL-22 in the organoid modality for 5 days and found there was a significant increase in both the lysozyme+ and DEFA5+ population of cells (Figure 2*A*). Furthermore, although there were no changes in the expression of *DEFA5* and *DEFA6,* there were significant increases in the expression of *LYZ* and *PLA2G2A* (Figure 2*B*), thereby demonstrating their responsivity to cytokines.

As Paneth cell responses to microbial ligands/microbes are of considerable interest, we aimed to investigate such responses. We first investigated for the presence of various bacterial sensors in enriched Paneth cell eHIOs and confirmed the presence of TLR2, TLR5, and NOD2 (Figure 2*C*). As intestinal organoids are polarized towards the lumen, we dissociated enriched organoids and seeded them onto Transwells, whereby the luminal aspect could be accessed as previously described,[8] so as to examine the effects of both microbial ligands and live bacteria and also quantify the secreted AMPs via enzyme-linked immunosorbent assay and mass spectrometry. We confirmed that Paneth cell markers remained highly enriched after seeding onto Transwells, indicating these cells were retained upon seeding (Supplementary Figure 2*C*) and also suggests the additional secretory cell types were present (Supplementary Figure 2*D*). We tested for ligands of the aforementioned receptors and found that the NOD2 ligand MDP caused a significant increase in the secretion of lysozyme (Figure 2*D*) but not by the Pam3CSK4, Flagellin, or LPS which is similar to a previous study.[9] Importantly, MDP did not induce lysozyme secretion in undifferentiated eHIOs, indicating that this response is specific to the Paneth cell-enriched state. Finally, we co-cultured live bacteria, obtained from resected ileocolonic tissue (for schematic see Figure 2*E*), in Transwells with Paneth cell-enriched monolayers and found, via mass spectrometry, a significant increase in DEFA5 secretion, among others (Figure 2*F* and *G*), which additionally underscores a Paneth cell-specific response. To identify pathways altered by bacterial exposure, we performed Gene Ontology (GO) Biological Process enrichment analysis using STRING (version 12.0) in significantly changed secreted proteins (false discovery rate [FDR] <0.05). Consistent with the analysis, "Antimicrobial humoral response" and "Defense response to bacterium" were among the most upregulated pathways (Figure 2*H*).

Our goal was to enrich the Paneth cell population in eHIOs generated from iPSCs, and we demonstrate that we have achieved this aim by illustrating the presence of AMPs and their responses to various stimuli.

Figure 2. (***See previous page***). **Functional responses of iPSC-derived Paneth cells.** (*A*) Representative flow cytometry dot plots of lysozyme+ and DEFA5+ cells in eHIOs cultured with ENC(3)+D and treated either with/without IL22 (10 ng/mL) with an accompanying graph showing data from 7 independent experiments. (*B*) qRT-PCR of Paneth cell-related genes in ENC(3)+D-treated eHIOs with/without IL22 (10 ng/mL). Student's *t*-test *$P < .05$; **$P < .01$ as compared with no addition of IL-22. Data from 4 independent experiments. (*C*) Representative fluorescent images showing enriched Paneth cell eHIOs are immunopositive for TLR2, TLR5, and NOD2 (*all red*), lysozyme (*green*), and E-cadherin (*white*). Scale bar is 50 μm. (*D*) Secretion of lysozyme in Paneth cell-enriched monolayers in response to apical administration of MDP, LPS, Pam3CSKJ4, and Flagellin. Undifferentiated monolayers were included as control (*right panel*). ***$P < .001$ as compared with untreated control. Data from 3 to 4 independent experiments. (*E*) Schematic of studies utilizing microbial cultures obtained from patients with inflammatory bowel disease. (*F*) Heatmap of differentially secreted proteins in media obtained from Paneth cell-enriched monolayers collected following 30-minute exposure to bacterial enriched suspensions derived from Crohn's disease mucosal scrapings. Samples are grouped as Treated (bacteria-exposed) or Untreated (control). Data represent mean values from 3 independent experiments. (*G*) Volcano plot of differential protein expression between Treated and Untreated secretome samples. Log_2 fold change (log_2FC) is plotted against $-log_{10}$ adjusted *P* value (false discovery rate [FDR]). Proteins meeting significance thresholds (FDR < 0.05, $|log_2FC| > 1$) are labeled. Data represent combined results from 3 independent experiments. (*H*) Gene Ontology (GO) Biological Process enrichment analysis of proteins significantly altered in the secretome following bacterial treatment. Terms are ranked by enrichment significance ($-log_{10}$ FDR). Circle size reflects the number of associated proteins, and color indicates FDR. Analysis performed using STRING (version 12.0) based on data from 3 independent experiments.

Given that iPSCs can be generated from almost all individuals, this now permits a methodology whereby Paneth cells from a range of individuals, including those with Crohn's disease, among others, can be generated and thus allows for studies into how genetic variations, microbial ligands/microbes, and components of the mucosal immune system influence this cell type.

SHACHI PATEL[*]
MONICA S. WAGNER[*]
OLIVIA BAY
CHRISTIAN E. WONG VALENCIA
ELISKA ZGARBOVA
Cedars-Sinai Medical Center
Board of Governors Regenerative Medicine Institute
Los Angeles, California

CYNTHIA I. RODRIGUEZ
Cedars-Sinai Medical Center
F. Widjaja Inflammatory Bowel Disease Institute
Los Angeles, California

DANIEL N. LEAL
MICHIFUMI YAMASHITA
Department of Pathology and Laboratory Medicine
Cedars-Sinai Medical Center
Los Angeles, California

SUZANNE DEVKOTA
Cedars-Sinai Medical Center
F. Widjaja Inflammatory Bowel Disease Institute
Los Angeles, California, *and*
Department of Medicine
Cedars-Sinai Medical Center
Human Microbiome Research Institute
Los Angeles, California, *and*
Department of Medicine and Biomedical Sciences
Cedars-Sinai Medical Center
Los Angeles, California

KATHRIN S. MICHELSEN
STEPHAN R. TARGAN
Cedars-Sinai Medical Center
F. Widjaja Inflammatory Bowel Disease Institute
Los Angeles, California, *and*
Department of Medicine and Biomedical Sciences
Cedars-Sinai Medical Center
Los Angeles, California

ROBERT J. BARRETT
Cedars-Sinai Medical Center
Board of Governors Regenerative Medicine Institute
Los Angeles, California, *and*
Cedars-Sinai Medical Center
F. Widjaja Inflammatory Bowel Disease Institute
Los Angeles, California, *and*
Department of Medicine and Biomedical Sciences
Cedars-Sinai Medical Center
Los Angeles, California

Supplementary Material

Note: To access the supplementary material accompanying this article, visit the full text version at https://doi.org/10.1016/j.jcmgh.2026.101769.

References

1. Cadwell K, et al. Nature 2008;456:259–263.
2. VanDussen KL, et al. Gastroenterology 2014;146:200–209.
3. Shi Y, et al. Nat Rev Drug Discov 2017;16:115–130.
4. Barrett R, et al. Stem Cells Transl Med 2014;3:1429–1434.
5. Tsai YH, et al. Development 2017;144:1045–1055.
6. Workman MJ, et al. Cell Mol Gastroenterol Hepatol 2018;5:669–677.e2.
7. Burclaff J, et al. Cell Mol Gastroenterol Hepatol 2022;13:1554–1589.
8. Gleeson JP, et al. Int J Mol Sci 2020;21:1438.
9. Farin HF, et al. J Exp Med 2014;211:1393–1405.

***Authors share co-first authorship.**

Most current article

© 2026 The Authors. Published by Elsevier Inc. on behalf of American Gastroenterological Association Institute. This is an open access article under the CC BY license (http://creativecommons.org/licenses/by/4.0/).
2352-345X
https://doi.org/10.1016/j.jcmgh.2026.101769

Received September 12, 2025. Accepted March 10, 2026.

Correspondence
Address correspondence to: Robert J. Barrett, PhD, Board of Governors Regenerative Medicine Institute, F. Widjaja Foundation Inflammatory Bowel Disease Institute, Cedars-Sinai Medical Center, Advanced Health Sciences Pavilion, Room 8308, 127 South San Vincente Boulevard, Los Angeles, California 90048. e-mail: Robert.Barrett@csmc.edu.

Acknowledgments
The authors wish to thank the Cedars-Sinai Biomanufacturing Center iPSC Core for the provision of the iPSC lines utilized in this study. The authors also wish to thank the Cedars-Sinai Proteomics and Metabolomics Core for their assistance with the mass spectrometry analysis and this manuscript.

Conflicts of interest
These authors disclose the following: Shachi Patel, Monica Silveira Wagner, Stephan R. Targan and Robert J. Barrett are named as inventors on a United States Patent Application No. 19/458,165, titled "Methodologies to Generate Human Paneth Cells and Enterochromaffin Cells and Determine Their Responses." The remaining authors disclose no conflicts.

Funding
This study is supported by the National Institutes of Health R01 DK123511 (Stephen Targan) and the F. Widjaja Inflammatory Bowel Disease Institute. Monica Wagner and Christian Wong Valencia were supported by the California Institute of Regenerative Medicine (CIRM-EDUC4-12751). The study sponsors played no role in the study design collection, analysis, or interpretation of data.

Cellular and Molecular Gastroenterology and Hepatology, Volume *20*

Supplemental information

A Method to Enrich Functional Human Paneth Cells in Induced Pluripotent Stem Cell-Derived Intestinal Organoids

Shachi Patel, Monica S. Wagner, Olivia Bay, Christian E. Wong Valencia, Eliska Zgarbova;Cynthia I. Rodriguez;Daniel N. Leal, Michifumi Yamashita;Suzanne Devkota;Kathrin S. Michelsen, Stephan R. Targan;, and Robert J. Barrett

Supplementary data

Supplementary Figure 1

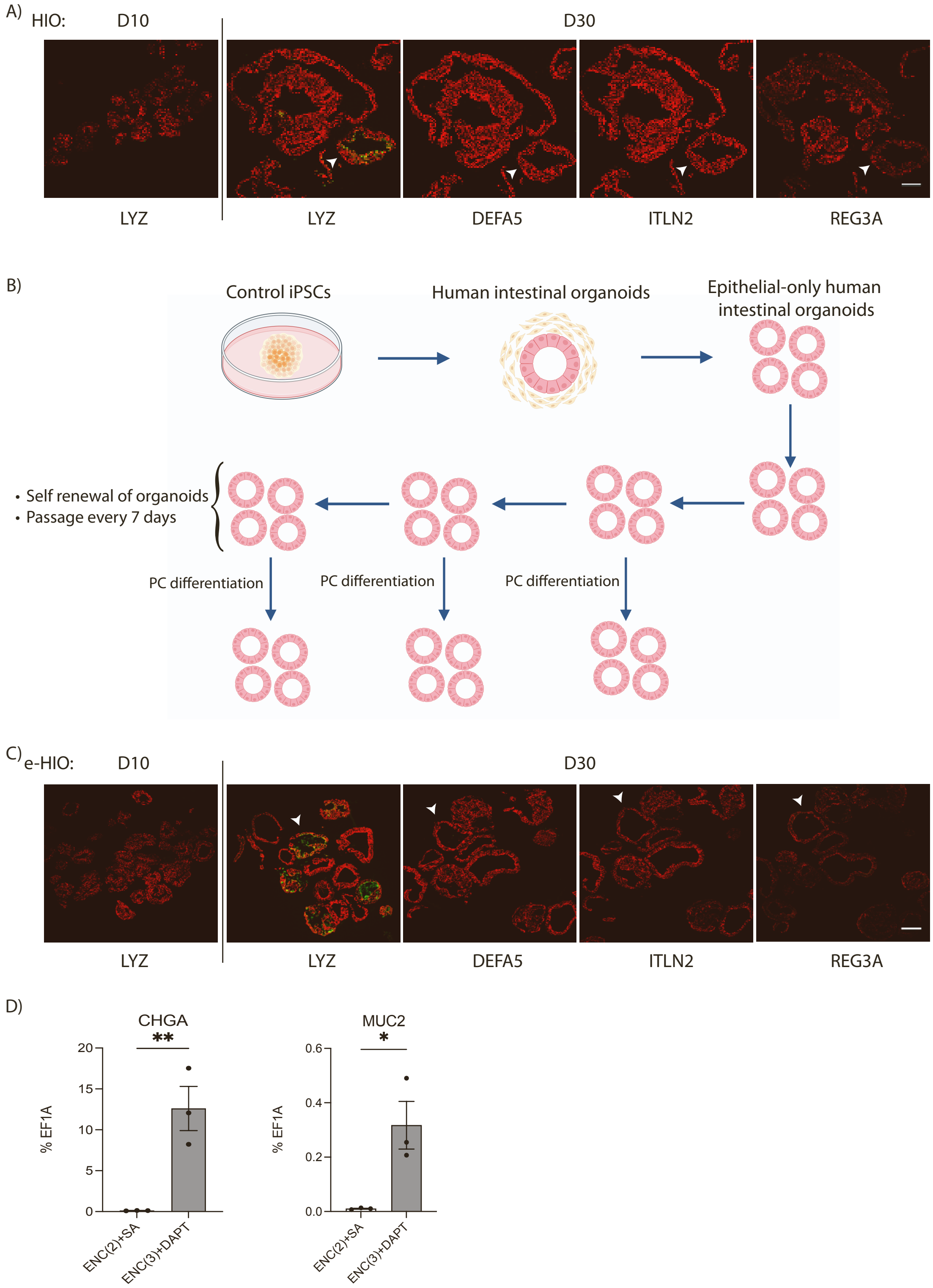

Supplementary Figure 1: Characterization of HIOs and eHIOs. (A) Representative fluorescent images of 10d and 30d HIOs immunopositive for E-cadherin (red) and lysozyme, DEFA5, REG3A and ITLN2 (green). Scale bar = 100μm. B) Schematic illustrating the generation of eHIOs from iPSCs. C) Representative fluorescent images of 10d and 30d e-HIOs immunopositive for E-cadherin (red) and lysozyme, DEFA5, REG3A and ITLN2 (green). Scale bar = 50μm. D) qPCR analysis of *CHGA* and *MUC2* expression in eHIOS cultured in ENC(2)+SA or ENC(3)+DAPT. Data represent mean ± SEM from 3 biological replicates. Students *t* test: **P* < .05 ***P* < .01 versus ENC(2)+SA.

Supplementary Figure 2.

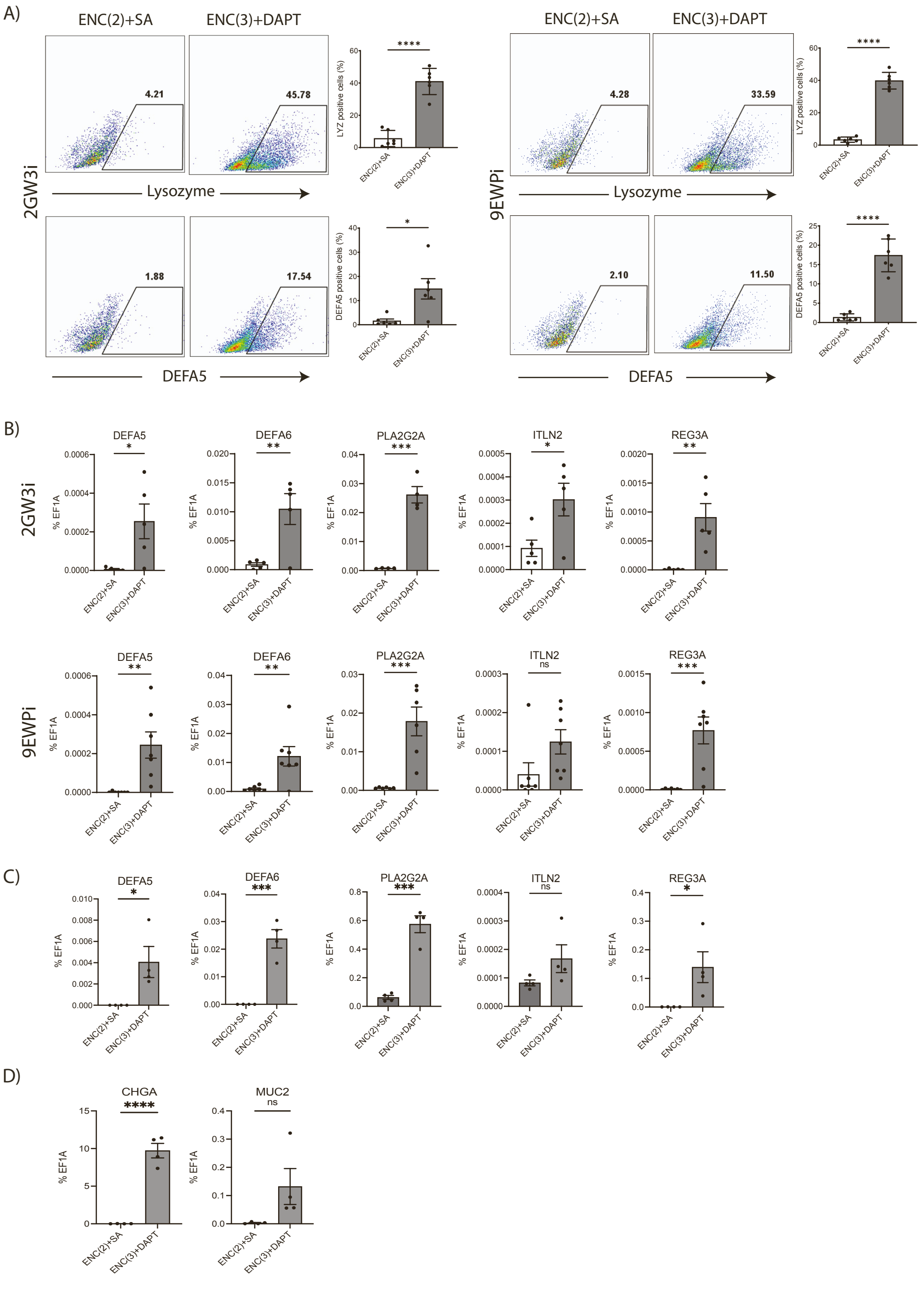

Supplementary Figure 2: Characterization of an enriched Paneth cell population in iPSC-derived eHIOs from 2 additional control lines, along with characterization of Paneth cell-enriched transwell cultures.

A) Representative flow cytometry dot plots showing lysozyme^{+} and DEFA5^{+} cells in eHIOs generated from control lines 2GW3i and 9EWPi, cultured in ENC(2)+SA or ENC(3)+DAPT media with an accompanying quantification graph from 6 independent experiments. B) qRT-PCR analysis of Paneth cell-related genes (*DEFA5*, *DEFA6*, *PLA2G2A*, *ITLN2*, and *REG3A*) in eHIOs from each control line cultured in ENC(2)+SA or ENC(3)+DAPT. (C) qRT-PCR characterization of Paneth cell markers **(***DEFA5*, *DEFA6*, *PLA2G2A*, *ITLN2*, *REG3A***)** in Paneth cell–enriched monolayers seeded on Transwell inserts and cultured in ENC(2)+SA or ENC(3)+DAPT. D) qRT-PCR analysis of enteroendocrine (*CHGA*) and goblet cell (*MUC2*) markers in Paneth cell-enriched transwell cultures maintained in ENC(2)+SA or ENC(3)+DAPT. Each value represents Mean ± SEM. Students *t* test *$P < .05$, **$P < .01$ and ***$P < .001$ as compared to ENC(2)+SA media.

Supplemental Table 1

Source/Specie	Proliferation media	Differentiation media	Time differentiating	Change in PC		Reference
				Mouse	Human	
Mouse small-intestinal crypts	EGF, Noggin, R-spondin1 (conditioned medium 5%final volume)	CHIR (2uM) and R-spondin1 (conditioned medium 10-20% final volume)	4 days	Immunostaining: increased LYZ^{+}; Number of PC/bud: increased	——	[1]
Mouse and human small-intestinal crypts	EGF, Noggin, R-spondin1, CHIR99021, Valproic acid	CHIR99021 (3uM) and DAPT (10uM)	4-7 days	Immunostaining: Day 4: 47% LYZ+ cells; Day 7: 85% LYZ+ cells; qPCR: ~15–20 fold Lyz1.	qPCR: ~3 fold Lyz	[2]
Human small-intestinal biopsies (upper and lower endoscopy)	50% conditioned media (Wnt3a, R-spondin, Noggin) + 50% fresh primary culture medium supplemented with Y-27632 and SB431542	5% or 50% of conditioned media (Wnt3a, R-spondin, Noggin) and DAPT (10uM)	2 -3 days	——	qPCR: 8–10 fold DEFA5 (ileal), 25–30 fold DEFA5 (duodenal).	[3]
Mouse small-intestinal crypts	EGF, Noggin, R-spondin1, CHIR99021, Valproic acid	CHIR (3uM) and DAPT (10uM)	2, 4 and 6 days	Immunostaining: 20–30% LYZ^{+}/DEFA^{+} cells. qPCR: ~5–7 fold Lyz1, ~5–7 fold Mmp7, ~2.5–6 fold Defa1. Proteomics: upregulation of AMPs including DEFA (10), CRS peptides (5), ribonucleases (6), lectins (12), Lyz1, and Pla2g1b. Secretion: increased LYZ activity. Bacteria growth: suppresses growth of L. lactis (strain MG1363).	——	[4]
Mouse small-intestinal crypts	EGF, Noggin, R-spondin1	CHIR99021 (3uM) and DAPT (10uM)	2-7 days	RNA-seq / transcriptomics: Upregulation of Lyz1, Ang4, Reg3γ, Pla2g2a, and Defa2	——	[5]
Mouse small-intestinal crypts; adult human duodenum tissue	EGF, Noggin, R-spondin1, CHIR99021, Valproic acid	DAPT (10uM) and KPT-330	6 days	Immunostaining: increased LYZ^{+} cells; Flow Cytometry: ~20% LYZ^{+}/CD24^{+}cells; LYZ Secretion: increased.	Immunostaining: increased LYZ^{+} cells; LYZ Secretion: increased. scRNA-seq: increased LYZ expression.	[6]
Human small-intestinal crypts (ileum, duodenum)	EGF, Noggin, R-spondin, A-83-01, WNT surrogate (1:1000), CHIR99021	Removal of CHIR99021, WNT surrogate (1:30000), IL-22 (2ng/mL)	7 days	——	Immunostaining: 1–3% DEFA5+ cells. qPCR: increased DEFA5, DEFA6, REG3G. FACS: increased DEFA5+ cells. scRNA-seq: upregulation of DEFA6, PLA2G2A, PRSS2, REG3A, ITLN2, LYZ.	[7]
Mouse small-intestinal crypts; human terminal ileum	IntestiCult Organoid Growth Medium (StemCell Technologies, #06010)	IntestiCult Organoid Differentiation Medium (Stem Cell Techologies, #100-02140) + AS1842856 (1uM)	2 days	——	Immunostaining: DEFA5+ cells. qPCR: incresead DEFA5, DEFA6, REG3A, PLA2G2A, ITLN2.	[8]
Human small-intestinal biopsies	EGF, IGF-1, FGF-2, DMH1, Noggin, R-spondin, Gastrin I, A-83-01, CHIR99021, Trichostatin A (TSA), 2-phospho-L-ascorbic acid (pVc), CP673451	Removal of TSA and CP6773451 and addition of DAPT (10uM) and IL-22 (2ng/mL)	3 days	——	Immunostaining: 15% LYZ^{+} and DEFA5 (not specified)	[9]

Supplemental Table 2

Media	Duration	Component	Final Concentration	Supplier / Catalog No.	Notes
**1. Organoid Medium (HIO Media)** Base formulation used pre-MACS +A2:F14sort	Passaged every 7–10 days prior to MACS sort. (Culture duration: 30 days)	Adv DMEM/F12	—	Gibco, 12634-010	Base medium
		B-27 Supplement	1×	Gibco, 17504-044	Added at all stages
		CHIR99021	2 µM	Tocris, 4423	Wnt activation; maintained through HIO culture
		Noggin	100 ng/mL	R&D Systems, 6057-NG	BMP inhibition
		EGF	100 ng/mL	R&D Systems, 236-EG	Supports epithelial growth
		L-glutamine	1% v/v	Gibco	Metabolic support
		Penicillin-Streptomycin	1% v/v	Gibco	Antimicrobial
**2. Epithelial-only Organoid Media (eHIO Media)** Base formulation used *post-MACS sort*	Passaged every 7 days as part of cell culture maintenance. Media refreshed every 2 days	HIO Media	—	—	Base formulation
		SB202190	10 µM	Tocris, 1264	p38 inhibitor; add fresh at each medium change
		A83-01	500 nM	Tocris, A83-01	TGF-β inhibitor; add fresh at each medium change
**3. Paneth Cell Enrichment Media (PC Media)** Base formulation used for Paneth Cell differentiaion	**1.** Culture single epithelial cells in matrigel domes for 2 days in eHIO media; **2.** On 3rd day switch to PC media for 5 days. **3.** Split developed organoid structure into new matrigel domes from1 to 2 parts and culture in PC media for another 5 days (no single cell dissociation); Media refreshed every 2 days. (Culture duration: 12days; 2 days eHIO media +10 days PC Media)	HIO Media (as above)	—	—	Base formulation
		CHIR99021	3 µM	Tocris, 4423	Wnt activator; add fresh at each medium change
		DAPT	10 µM	R&D Systems, 2634/10	Notch inhibitor; add fresh at each medium change; drives Paneth cell differentiation

Supplemental Table 2. Media formulations used for HIO and eHIO culture and Paneth cell enrichment. This table lists all components, final concentrations, suppliers, and catalog numbers for each media condition used in the study, including HIO maintenance media, epithelial-only HIO (eHIO) expansion media, and the modified ENC(3) and ENC(3)+DAPT formulations. Stage-specific notes are provided to enable full replication of the differentiation and enrichment protocol

Methods:

Statement of Ethics

All the cell lines and protocols in the present study were carried out in accordance with the guidelines approved by the stem cell research oversight committee and institutional review board at the Cedars-Sinai Medical Center under the auspice of the institutional review board stem cell research oversight committee protocols Pro00027264 and Pro0004857. All authors had access to the study data and had reviewed and approved the final manuscript.

Cell Lines and Culture Conditions

The CS03iCTR-n1, CS2GW3iCTR-n1, and CS9EWPiCTR-n1 iPSC lines were obtained from the iPSC Core at Cedars-Sinai Medical Center (Los Angeles, CA, USA) and were derived from healthy control subjects. These lines were fully characterized for pluripotency markers and confirmed to be karyotypically normal. iPSCs were maintained in an undifferentiated state on Matrigel®-coated plates (Corning, 354234; Corning, NY, USA) in mTeSR™1 medium (STEMCELL Technologies, 85851; Vancouver, BC, Canada) under feeder-free conditions. Cells were passaged every 4–5 days using ReLeSR™ (STEMCELL Technologies, 100-0483; Vancouver, BC, Canada).

HIO Generation from iPSCs

The generation of human intestinal organoids (HIOs) from iPSCs was performed using a multistep protocol as previously described [10]. Briefly, iPSCs were directed to definitive endoderm, followed by hindgut structures, and ultimately into organoids. All HIOs were cultured in medium containing CHIR99021 (2 µM; Tocris Bioscience, 4423; Bristol, UK), Noggin (100 ng/mL; R&D

Systems, 6057-NG; Minneapolis, MN, USA), EGF (100 ng/mL; R&D Systems, 236-EG; Minneapolis, MN, USA), B-27 Supplement (1×; Gibco, 17504-044; Waltham, MA, USA), and DMEM/F12 (Gibco, 12634-010; Waltham, MA, USA), supplemented with penicillin/streptomycin and L-glutamine (5% v/v). All organoid cultures were embedded in standard Matrigel® Basement Membrane Matrix (Corning 354234), a non–growth factor–reduced formulation used consistently across all experiments. Organoids were passaged every 7–10 days and subjected to magnetic-activated cell sorting (MACS) after 30 days of differentiation.

Generation of Purified Epithelial Cell Cultures

HIOs were removed from Matrigel and washed 3 times in Dulbecco's Phosphate-Buffered Saline (DPBS; Gibco, 14190-144; Waltham, MA, USA) and subsequently incubated in TrypLE™ Select (Gibco, 12563-029; Waltham, MA, USA) for 12 minutes until the organoids were completely dissociated to a single-cell suspension. Dissociated cells were incubated with EpCAM MicroBeads (Miltenyi Biotec, 130-061-101; Bergisch Gladbach, Germany) for 30 minutes at 4 °C. EpCAM$^+$ cells (eHIOs) were obtained via the autoMACS® Pro Separator (Miltenyi Biotec, Bergisch Gladbach, Germany). Following isolation, EpCAM$^+$ epithelial cells were resuspended in Matrigel as single cells, where they self-organized and expanded to form epithelial-only HIOs (eHIOs). eHIOs were maintained in organoid medium supplemented with SB202190 (10 µM; Tocris Bioscience, 1264; Bristol, UK) and A83-01 (500 nM; Tocris Bioscience, A83-01; Bristol, UK).

Enrichment of Paneth Cells

eHIOs were split once a week, and the medium was refreshed every 3 days. For passaging, eHIOs were removed from Matrigel, washed 3 times with DPBS (Gibco, 14190-144; Waltham, MA, USA), and incubated in TrypLE™ Select (Gibco, 12563-029; Waltham, MA, USA) for 12 minutes until fully dissociated into a single-cell suspension. Cells were then seeded into fresh Matrigel domes and maintained in eHIO expansion medium for 2 days. After 2 days, the culture medium was replaced with differentiation medium containing increased CHIR99021 (3 µM; Tocris Bioscience, 4423; Bristol, UK), IL-22 (2ng/mL; R&D Systems, 782IL; Minneapolis, MN, USA) and DAPT (10 µM; R&D Systems, 2634/10; Minneapolis, MN, USA) to promote Paneth cell differentiation. Organoids were maintained in this differentiation medium for 5 days. Subsequently, the expanded eHIOs were replated without enzymatic dissociation and cultured for an additional 5 days in the same differentiation medium. On Day 12 of the differentiation protocol, cells were harvested and analyzed for Paneth cell enrichment. To assess Paneth cell antimicrobial function at the transcriptional level, eHIOs were stimulated with IL-22 (10 ng/mL; R&D Systems, 782IL; Minneapolis, MN, USA) for 72 or 120 hours, starting on days 10 and 12 of the differentiation protocol, respectively.

Quantitative Polymerase Chain Reaction (qPCR)

RNA was extracted from eHIOs using the RNeasy® Mini Kit (Qiagen, 74106; Hilden, Germany). Complementary DNA (cDNA) was synthesized from 500 ng of RNA using the High-Capacity cDNA Reverse Transcription Kit (Applied Biosystems™, 4368814; Foster City, CA, USA). Quantitative real-time PCR was performed using SYBR™ Select Master Mix (Applied Biosystems™, 4472908;

Foster City, CA, USA) on a Bio-Rad CFX384 Real-Time System (Bio-Rad Laboratories, Hercules, CA, USA). Primer sequences are listed in

Table 1.

Gene name	Forward Primer	Reverse Primer
EF1A	TATTGGATTGCCACACGGCT	AAAGCGACCCAAAGGTGGAT
LYZ	TTTCTGTTACGGTCCAGGGC	ACACATCCAGTTTGCTAGGCT
DEFA5	TGGGGAAGACAACCAGGAC	TTCGGCAATAGCAGGTGG
DEFA6	GAGGATGCAAGCTCAAGTCTTAG	TGCAATGGCAAGTGAAAGC
REG3A/G	ACTGCTATGCCTTGTTTTTGTC	CTACTCCACTCCCAACCTTCTC
SPLA2G2A	CGCACTCAGTTATGGCTTCTAC	GTGATTCTGCTCCCCGAG
ITLN2	CTCCTTTTCTTCCCTGCCTAG	TCTGGTAGACAACACCATTCTTG
PRSS2	CCCTCATCAGCGAACAGTG	CAGGATGTCATTGTCCAGAGTC
TLR2	CGGCGTTCTCTCAGGTGA	TGGCATTGTCCAGTGCTTC
TLR5	TGCTACTGACAACGTGGCTTC	ACATCTGAGGCTCCGACATC
NOD2	GTGGAGAACATGCTGGACCT	GCCAATGGGACTGGTAATTC

Immunocytochemistry and Microscopy

eHIOs were fixed in 4% paraformaldehyde (Electron Microscopy Sciences, 15714-5; Hatfield, PA, USA), transferred to 30% sucrose overnight at 4 °C, embedded in Tissue-Tek® O.C.T. Compound (Sakura Finetek, 4583; Torrance, CA, USA), and cryosectioned at 8 μm thickness. Sections were blocked with 10% normal donkey serum in 0.5% Triton X-100 and incubated overnight at 4 °C with primary antibodies (Table 2). The following day, sections were rinsed and incubated with Alexa Fluor 488 (1:1000; Invitrogen, A21202; Carlsbad, CA, USA), Alexa Fluor 594 (1:1000; Invitrogen, A32758; Carlsbad, CA, USA) or Alexa Fluor 647 (1:1000; Invitrogen, A21447; Carlsbad, CA, USA) for 1 hour at room temperature, followed by DAPI counterstaining (1:5000; Invitrogen, H3570; Carlsbad, CA, USA). Slides were imaging using Nikon Eclipse Ti- A1R HD.

Antigen	Host Species	Manufacturer	Catalog number	Dilution
Lysozyme	Mouse	Novus Biologicals	NB100-63062	1/500
Lysozyme	Rabbit	Invitrogen	PA5-16668	1/250
NOD2	Mouse	Novus Biologicals	NB100-524	1/100
DEFA5	Mouse	Novus Biologicals	NB110-60002	1/1250
ITLN2	Mouse	R&D Systems	MAB8004	1/100
Reg3A	Mouse	R&D Systems	MAB5965	1/200
E-cadherin	Goat	R&D Systems	AF648	1/1000
TLR2	Rabbit	Novus Biologicals	NB100-56720	1/250
TLR5	Mouse	Novus Biologicals	NBP2-24787	1/250

Organoid Epithelial Cell Monolayer

Paneth cell-enriched eHIOs were dissociated into single cells using TrypLE™ Select (Gibco, 12563-029; Waltham, MA, USA) and seeded onto 6.5 mm Transwell® inserts (STEMCELL Technologies, 38024; Vancouver, BC, Canada) pre-coated with Matrigel® (Corning, 354234; Corning, NY, USA) at a density of 4.0×10^5 cells per well. Cells were cultured in differentiation medium for 24 hours to allow monolayer formation and stabilization prior to stimulation.

Enzyme-Linked Immunosorbent Assay (ELISA)

Media from transwell inserts were collected after exposure to LPS (100 µg/mL; Sigma-Aldrich, L2880; St. Louis, MO, USA), Pam3CSK4 (10 µg/mL; Novus Biologicals, NBP2-25297; Centennial, CO, USA), and MDP (100 µg/mL; InvivoGen, tlrl-mdp; San Diego, CA, USA) for 30 minutes. Human lysozyme ELISA (Novus Biologicals, NBP2-60511; Centennial, CO, USA) was performed according to the manufacturer's instructions. Absorbance at 450 nm and 570 nm was measured using a SpectraMax M3 microplate reader (Molecular Devices, San Jose, CA, USA).

Flow Cytometry

eHIOs were removed from Matrigel, washed three times with DPBS (Gibco, 14190-144; Waltham, MA, USA), and incubated in TrypLE™ Select (Gibco, 12563-029; Waltham, MA, USA) for 12 minutes at 37 °C to generate a single-cell suspension. Dissociated cells were passed through a 70 μm mini cell strainer (pluriSelect, 43-10070-40; Leipzig, Germany) to remove aggregates and debris. Cells were then fixed in Flow Cytometry Fixation Buffer (R&D Systems, FC004; Minneapolis, MN, USA) and permeabilized using Flow Cytometry Permeabilization/Wash Buffer (R&D Systems, FC005; Minneapolis, MN, USA), following the manufacturer's protocol. Permeabilized cells were incubated with unconjugated primary antibodies: anti-Lysozyme (1:100; Novus Biologicals, NB100-63062; Centennial, CO, USA) and anti-DEFA5 (1:2000; Novus Biologicals, NB110-60002; Centennial, CO, USA) for 30 minutes at 4 °C. After three washes, cells were incubated with Alexa Fluor 488-conjugated secondary antibody (1:100; Invitrogen, A21202; Carlsbad, CA, USA) for 30 minutes at 4 °C in the dark. Samples were analyzed using a BD LSR Fortessa™ Cell Analyzer (BD Biosciences, San Jose, CA, USA), and data were analyzed with FlowJo software (BD Biosciences, San Jose, CA, USA).

Transmission Electron Microscopy

Organoid samples were fixed in 2.5% glutaraldehyde and 2% formaldehyde in 0.1 M sodium cacodylate at 4 ˚C. After fixation, samples were dehydrated and embedded in Epon 812 (Polysciences). The ultrathin sections were stained with 1% uranyl acetate and 3% lead citrate. Ultimately, the images were taken using HT7700 transmission electron microscope (HITACHI) at 1,200x direct magnification.

Bacterial Isolation from Crohn's Disease Intestinal Resections

Intestinal tissue specimens were collected aseptically from patients with Crohn's disease undergoing ileocolonic resections. Immediately following surgical excision, tissues were placed in sterile containers and transferred from the operating room to a biosafety cabinet for processing within 1 h of resection. Tissues were rinsed with sterile DPBS (Gibco, 14190-144; Waltham, MA, USA) until all visible surface blood was removed. Sutured ends were excised, and the bowel was opened longitudinally. Mucosal scrapings were obtained, yielding 200–500 µL of mucosal material per specimen, which was transferred into sterile microcentrifuge tubes. Mucosal scrapings were immediately placed in an anaerobic chamber (Coy Laboratory Products, 602000; Grass Lake, MI, USA) maintained at 90% N_2, 5% CO_2, and 5% H_2. Samples were homogenized in 1 mL of reduced sterile DPBS and an aliquot (100 µL) of the homogenate was inoculated into pre-reduced 9.9 mL brain heart infusion (BHI; Sigma-Aldrich, B21202; St. Louis, MO, USA) and 9.9 mL Gifu Anaerobic Medium (GAM; HiMedia, M189; Mumbai, India). Cultures were incubated anaerobically at 37 °C for ≥12 h. Overnight cultures from both media were pooled, and bacterial suspensions were normalized to an optical density at 600 nm (OD_{600}). TW was treated with the pooled bacterial suspension at a final concentration of OD_{600} = 0.5 for 30 min, after which the media was collected for analysis.

Proteomics Sample Digest

Samples were digested using the SP3 protocol. Briefly, 20 µg of protein was brought to 60 µL with lysis buffer containing 6 M urea, 1 M ammonium bicarbonate, and 5% SDS (Thermo Fisher, 20230; Waltham, MA, USA). Proteins were reduced with 16.8 µL of 200 mM dithiothreitol (DTT;

Sigma-Aldrich, D9779; St. Louis, MO, USA) for 30 min at 37 °C with shaking at 300 rpm, then alkylated with 21.2 µL of 400 mM iodoacetamide (IAA; Sigma-Aldrich, I1149; St. Louis, MO, USA) at room temperature for 30 min in the dark. The volume was brought to 160 µL with Tris-HCl pH 8 (Thermo Fisher, AM9856; Waltham, MA, USA), and 5 µL of bead suspension (10:1 mass ratio of beads to protein; 1:1 mixture of hydrophilic/hydrophobic beads; Cytiva, 29152179; Marlborough, MA, USA) was added and vortexed. Samples were adjusted to 70% acetonitrile (ACN; Sigma-Aldrich, 34851; St. Louis, MO, USA) v/v and incubated for 18 min. Solvent was removed on-magnet, and samples were washed with 2× 80% ethanol (EtOH; Sigma-Aldrich, 459844; St. Louis, MO, USA) and 2× ACN (200 µL each). After complete solvent removal, samples were resuspended in 50 mM Tris-HCl pH 8 with 10 mM $CaCl_2$ (Sigma-Aldrich, C1016; St. Louis, MO, USA) and digested with trypsin (1:20 enzyme:protein; Promega, V5111; Madison, WI, USA). Samples were bath-sonicated for 5 min, then incubated for 18 h at 37 °C with shaking at 1200 rpm. After digestion, samples were removed from beads and brought to 0.1% formic acid (FA; Sigma-Aldrich, F8775; St. Louis, MO, USA) and 2% DMSO (Sigma-Aldrich, 276855; St. Louis, MO, USA) for LC-MS/MS injection.

LC-MS/MS Analysis

Approximately 500 ng of peptides from digested samples were analyzed on a Thermo Orbitrap Astral mass spectrometer (Thermo Fisher, A12345; Waltham, MA, USA) coupled to a NeoVanquish LC (Thermo Fisher, NLC123; Waltham, MA, USA). A blank injection was included after every three samples to assess carryover. Peptides were separated using a 24 min gradient with solvent A (0.1% formic acid in water; Sigma-Aldrich, F8875; St. Louis, MO, USA) and solvent

B (80% acetonitrile, 0.1% formic acid; Sigma-Aldrich, 34851; St. Louis, MO, USA) at 1.2 µL/min. The gradient was: 0 min, 4% B; 2 min, 9% B; 13 min, 25% B; 17 min, 35% B. LC was performed in direct injection mode using a PepSep C18 column (150 µm ID × 15 cm, 1.5 µm; Bruker, 100367; Billerica, MA, USA) coupled to a nano source (Thermo EasySpray, ES081; Waltham, MA, USA). MS data were acquired in data-independent acquisition (DIA) mode from 380–980 Da with 240k Orbitrap resolution, 10 ms maximum injection time for MS1, and 4 ms maximum injection time for MS2 with 4 Th windows.

Data Analysis

MS raw data files were searched against UniProt human reviewed protein sequence entries (accessed April 2023) using DIA-NN (v 1.8.1) [11] in library free mode with default parameters. Based on recent comparisons with library-based approaches, DIA-NN in library-free mode has been found to produce results that are comparable or better than those of experimental library-based searches while being freely available and was hence chosen for the analysis of all data (PMID: 36609502). The output protein group matrix from DIA-NN was used to perform downstream analysis using MetaboAnalyst 6.0 [12].

Gene ontology enrichment analysis

Proteins identified in the secretome were filtered for statistical significance using a p-value < 0.05 and false discovery rate (FDR) < 0.05. The filtered list of proteins was submitted to STRING (version 12.0; https://string-db.org) for functional enrichment analysis of Gene Ontology (GO) Biological Processes, using *Homo sapiens* as the reference species. STRING calculates enrichment

using a hypergeometric test and corrects for multiple comparisons with the Benjamini–Hochberg procedure. Enrichment results are reported as STRING-derived FDR values.

Statistical Analysis

All data are presented as mean ± standard error of the mean (SEM). Statistical analyses were performed using GraphPad Prism version 9.2.0 (GraphPad Software, San Diego, CA, USA). Comparisons among multiple groups were conducted using two-way analysis of variance (two-way ANOVA) followed by appropriate post hoc tests. Unpaired or paired t-tests were used for comparisons between two groups, as appropriate. A p-value $< .05$ was considered statistically significant.

Supplementary References:

[1] Farin HF, Van Es JH, Clevers H. Redundant sources of Wnt regulate intestinal stem cells and promote formation of Paneth cells. Gastroenterology 2012;143(6):1518-29 e7.

[2] Yin X, Farin HF, van Es JH, Clevers H, Langer R, Karp JM. Niche-independent high-purity cultures of Lgr5+ intestinal stem cells and their progeny. Nat Methods 2014;11(1):106-12.

[3] VanDussen KL, Marinshaw JM, Shaikh N, Miyoshi H, Moon C, Tarr PI, Ciorba MA, Stappenbeck TS. Development of an enhanced human gastrointestinal epithelial culture system to facilitate patient-based assays. Gut 2015;64(6):911-20.

[4] Mead BE, Ordovas-Montanes J, Braun AP, Levy LE, Bhargava P, Szucs MJ, Ammendolia DA, MacMullan MA, Yin X, Hughes TK, Wadsworth MH, 2nd, Ahmad R, Rakoff-Nahoum S, Carr SA, Langer R, Collins JJ, Shalek AK, Karp JM. Harnessing single-cell genomics to improve the physiological fidelity of organoid-derived cell types. BMC Biol 2018;16(1):62.

[5] Treveil A, Sudhakar P, Matthews ZJ, Wrzesinski T, Jones EJ, Brooks J, Olbei M, Hautefort I, Hall LJ, Carding SR, Mayer U, Powell PP, Wileman T, Di Palma F, Haerty W, Korcsmaros T. Regulatory network analysis of Paneth cell and goblet cell enriched gut organoids using transcriptomics approaches. Mol Omics 2020;16(1):39-58.

[6] Mead BE, Hattori K, Levy L, Imada S, Goto N, Vukovic M, Sze D, Kummerlowe C, Matute JD, Duan J, Langer R, Blumberg RS, Ordovas-Montanes J, Yilmaz OH, Karp JM, Shalek AK. Screening for modulators of the cellular composition of gut epithelia via organoid models of intestinal stem cell differentiation. Nat Biomed Eng 2022;6(4):476-94.

[7] He GW, Lin L, DeMartino J, Zheng X, Staliarova N, Dayton T, Begthel H, van de Wetering WJ, Bodewes E, van Zon J, Tans S, Lopez-Iglesias C, Peters PJ, Wu W, Kotlarz D, Klein C, Margaritis T, Holstege F, Clevers H. Optimized human intestinal organoid model reveals interleukin-22-dependency of paneth cell formation. Cell Stem Cell 2022;29(9):1333-45 e6.

[8] Eng SJ, Nonnecke EB, de Lorimier AJ, Ali MR, Tsolis RM, Bevins CL, Ashwood P. FOXO inhibition rescues alpha-defensin expression in human intestinal organoids. Proc Natl Acad Sci U S A 2023;120(47):e2312453120.

[9] Yang L, Wang X, Zhou X, Chen H, Song S, Deng L, Yao Y, Yin X. A tunable human intestinal organoid system achieves controlled balance between self-renewal and differentiation. Nat Commun 2025;16(1):315.

[10] Workman MJ, Gleeson JP, Troisi EJ, Estrada HQ, Kerns SJ, Hinojosa CD, Hamilton GA, Targan SR, Svendsen CN, Barrett RJ. Enhanced Utilization of Induced Pluripotent Stem Cell-Derived Human Intestinal Organoids Using Microengineered Chips. Cell Mol Gastroenterol Hepatol 2018;5(4):669-77 e2.

[11] Demichev V, Messner CB, Vernardis SI, Lilley KS, Ralser M. DIA-NN: neural networks and interference correction enable deep proteome coverage in high throughput. Nat Methods 2020;17(1):41-4.

[12] Pang Z, Lu Y, Zhou G, Hui F, Xu L, Viau C, Spigelman AF, MacDonald PE, Wishart DS, Li S, Xia J. MetaboAnalyst 6.0: towards a unified platform for metabolomics data processing, analysis and interpretation. Nucleic Acids Res 2024;52(W1):W398-W406.
